# Preparation, characterization and application of MgFe_2_O_4_/Cu nanocomposite as a new magnetic catalyst for one-pot regioselective synthesis of β-thiol-1,4-disubstituted-1,2,3-triazoles†

**DOI:** 10.1039/d1ra01588e

**Published:** 2021-04-07

**Authors:** Ronak Eisavi, Kazhal Naseri

**Affiliations:** Department of Chemistry, Payame Noor University PO Box 19395-3697 Tehran Iran roonak.isavi@gmail.com

## Abstract

Magnesium ferrite magnetic nanoparticles were synthesized by a solid-state reaction of magnesium nitrate, hydrated iron(iii) nitrate, NaOH and NaCl salts and then calcined at high temperatures. In order to prevent oxidation and aggregation of magnesium ferrite particles, and also for preparing a new catalyst of supported copper on the magnetic surface, the MgFe_2_O_4_ was covered by copper nanoparticles in alkaline medium. Magnetic nanoparticles of MgFe_2_O_4_/Cu were successfully obtained. The structure of the synthesized magnetic nanoparticles was identified using XRD, TEM, EDS, FT-IR, FESEM and VSM techniques. The prepared catalyst was used in the three component one-pot regioselective synthesis of 1,2,3-triazoles in water. The various thiiranes bearing alkyl, allyl and aryl groups with terminal alkynes, and sodium azide in the presence of the MgFe_2_O_4_/Cu nanocatalyst were converted to the corresponding β-thiolo/benzyl-1,2,3-triazoles as new triazole derivatives. The effects of different factors such as time, temperature, solvent, and catalyst amount were investigated, and performing the reaction using 0.02 g of catalyst in water at 60 °C was chosen as the optimum conditions. The recovered catalyst was used several times without any significant change in catalytic activity or magnetic property.

## Abstract 2

A magnetically separable MgFe_2_O_4_/Cu nanocomposite was prepared and used as a novel heterogeneous catalyst for the synthesis of new β-thiol-1,4-disubstituted-1,2,3-triazoles from thiiranes.

## Graphical abstract

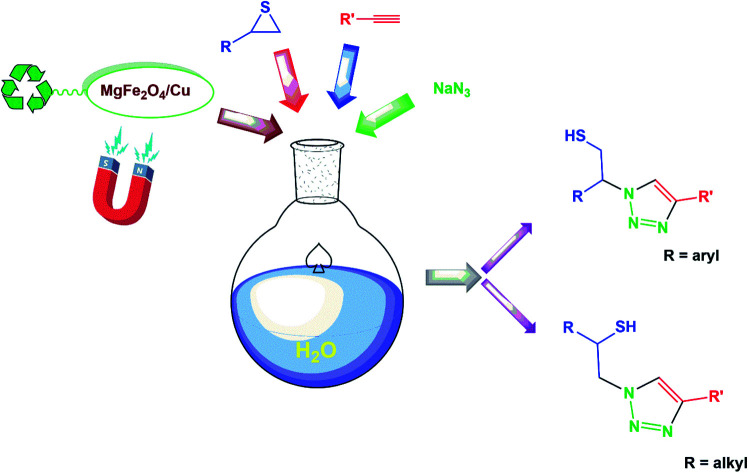

## 1. Introduction

In recent decades, 1,2,3-triazoles have attracted considerable attention due to their important biological and pharmacological activities such as analgesic,^1^ local anaesthetic,^2^ antimicrobial,^3,4^ anti HIV,^5^ anticancer,^6^ antiallergic,^7^ anticonvulsant,^8^ antiproliferative,^9^ antiviral, antitubercular,^10^ antifungal,^11^ antibacterial,^12^ antioxidants,^13^ antimalarial,^14^ and anti-inflammatory activities.^15^

Huisgen 1,3-dipolar cycloaddition between an azide and alkyne has rapidly gained importance for its potential to manufacture drug targets with different biological significance.^6^ Recently, one-pot synthesis of β-hydroxy-1,2,3-triazoles has been reported through the click reaction of azides, alkynes and epoxides catalyzed by various copper catalysts.^16–31^ Although nucleophilic ring opening of strained three-membered heterocycles such as epoxides and aziridines is easily carried out using different nucleophiles such as azides and amines, no cases have been reported yet for 1,3-dipolar cycloaddition between azide, alkyne and thiiranes.

Multi-component reactions (MCRs) are reactions in which three or more reactants react to generate only one product. MCRs present a convenient synthetic procedure for producing complex molecules with structural variety and molecular intricacy.^32^ These kinds of reactions provide major benefits like environmental compatibility, high efficiency, quick and plain performance, and reducing the reaction time and saving energy. Compared to conventional methods, these reactions require fewer steps to achieve the final product and can be performed in one-pot. Therefore, MCRs play significant roles in different research fields such as biomedical, synthetic organic, generating libraries of bioactive compounds, pharmaceutical and drug discovery research, industrial chemistry *etc.*^33–36^ An ideal multicomponent reaction permits the concurrent addition of all reactants, reagents and catalysts under the same reaction conditions. One-pot reactions show an efficient strategy in modern synthetic chemistry.^37^ Minimizing the number of synthetic steps in obtaining products from starting reactants is highly favorable in organic synthesis. The perfect regioselectivity and high purity of desired products, and excellent yields are among the other remarkable advantages of multicomponent one-pot reactions.

Ferrite nanoparticles due to their magnetic property are easily separable. Recently, they have received great attention in biomedicine,^38–40^ and organic synthesis.^41–44^ Nevertheless, the nano-ferrites have hydrophobic surfaces with a large surface to volume ratio and strong magnetic dipole–dipole attractions, and they always suffer from adsorption problems because of their intense tendency of self-aggregation and low quantity of functional groups.^45,46^ To prevent agglomeration of magnetic nanoparticles (MNPs) and improve their efficiency, surface coating of the MNPs is required.^47^ Aqueous MNP dispersions can be achieved by surface coating with copper nanoparticles.

**Scheme 1:**
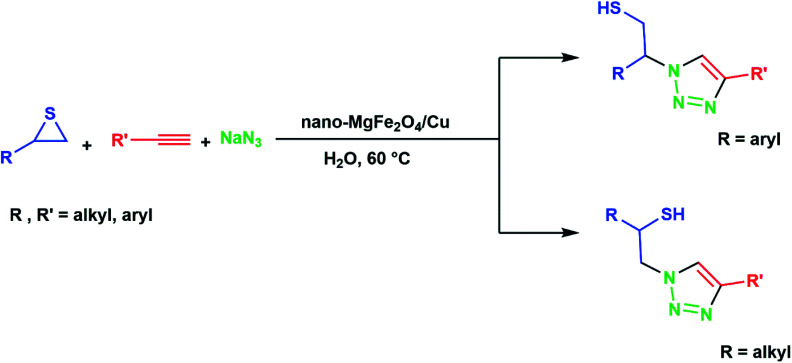
Synthesis of β-thiol-1,4-disubstituted-1,2,3-triazoles from thiiranes catalyzed by MgFe_2_O_4_/Cu.

In continuation of pioneering works on nano-ferrites,^48–54^ herein, we wish to report an efficient, three-component click reaction protocol for synthesis of β-thiol-1,4-disubstituted-1,2,3-triazoles as new triazole derivatives from sodium azide, thiiranes, and terminal alkynes in the presence of MgFe_2_O_4_/Cu magnetic nanoparticles as a novel and environmentally friendly heterogeneous catalyst in water (Scheme 1).

## 2. Experimental

### 2.1. Instruments and materials

All materials were purchased from the Merck and Aldrich Chemical Companies with the best quality and they were used without further purification. IR and ^1^H/^13^C NMR spectra were recorded on Thermo Nicolet Nexus 670 FT-IR and 500 MHz Bruker Avance spectrometers, respectively. Melting points were measured on an Electrothermal IA9100 microscopic digital melting point apparatus. The synthesized nanocatalyst was characterized by XRD on a Bruker D8-Advanced diffractometer with graphite-monochromatized Cu Kα radiation (*λ* = 1.54056 Å) at room temperature. TEM image was recorded using an EM10C-100 kV series microscope from the Zeiss Company, Germany. FESEM images were determined using FESEM-TESCAN. The energy dispersive X-ray spectrometer (EDS) analysis was taken on a MIRA3 FE-SEM microscope (TESCAN, Czech Republic) equipped with an EDS detector (Oxford Instruments, UK). Magnetic property of synthesized nanocatalyst was measured using a VSM (Meghnatis Daghigh Kavir Co., Kashan Kavir, Iran) at room temperature. HRMS analyses were also carried out in the electron impact mode (EI) at 70 eV. The Cu content on the catalyst was determined by Perkin Elmer Optima 7300DV ICP-OES analyzer.

### 2.2. Synthesis of MgFe_2_O_4_ nanoparticles

MgFe_2_O_4_ nanoparticles were synthesized by a solid-state procedure according to our reported investigation.^48^ Briefly, in a mortar, Mg(NO_3_)_2_·6H_2_O (0.512 g, 2 mmol), Fe(NO_3_)_3_·9H_2_O (1.61 g, 4 mmol), NaOH (0.64 g, 16 mmol), and NaCl (0.232 g, 4 mmol) were mixed in a molar ratio of 1 : 2 : 8 : 2 and ground together for 55 min. The reaction was carried out with the release of heat. After 5 minutes of grinding, the mixture became pasty and its color changed to dark brown. For removing the additional salts, the obtained mixture was washed with double-distilled water for several times. The produced mixture was dried at 80 °C for 2 h and it was then calcined at 900 °C for 2 h to obtain the MgFe_2_O_4_ nanoparticles as a dark brown powder.

### 2.3. Preparation of MgFe_2_O_4_/Cu nanocomposite

In a round-bottom flask, a solution of CuCl_2_·2H_2_O (0.68 g, 4 mmol) in distilled water (50 mL) was prepared and then MgFe_2_O_4_ (1 g) was added. The mixture was stirred vigorously for 30 min and followed by gradually addition of KBH_4_ powder (0.1 g) in order to reduce Cu^2+^ cations to copper nanoparticles. The stirring of mixture was continued at room temperature for 1 h. The black MgFe_2_O_4_/Cu nanocomposite was separated using a magnet, washed with distilled water and then dried under air atmosphere.

### 2.4. Solvent-free synthesis of thiiranes from epoxides: general procedure

The various thiiranes were prepared using a solvent-free method reported in our previous research.^55^ Briefly, a mixture of epoxide (1 mmol) and alumina immobilized thiourea (0.752 g, 25% w/w) was ground in a mortar for an appropriate time at room temperature. The progress of the reaction was monitored by TLC using *n*-hexane : EtOAc (5 : 2) as an eluent. After completion of the reaction, the mixture was washed with EtOAc (3 × 5 mL). The combined washing solvents were evaporated under reduced pressure to give the crude thiirane for further purification by a short-column chromatography over silica gel.

### 2.5. One-pot synthesis of β-thiol-1,4-disubstituted-1,2,3-triazoles from thiiranes catalyzed by MgFe_2_O_4_/Cu in water: a general procedure

In a round-bottomed flask equipped with a magnetic stirrer and condenser, a solution of the thiirane (1 mmol), alkyne (1 mmol) and sodium azide (0.078 g, 1.2 mmol) in H_2_O (5 mL) was prepared. MgFe_2_O_4_/Cu nanocomposite (0.02 g) was then added to the solution and the resulting mixture was stirred magnetically for 2–4 h at 60 °C. The progress of the reaction was monitored by TLC using *n*-hexane : EtOAc (10 : 2) as an eluent. After completion of the reaction, the magnetic nanocatalyst was separated using an external magnet and collected for the next run. The reaction mixture was extracted with ethyl acetate and then dried over anhydrous Na_2_SO_4_. After evaporating the organic solvent, the crude β-thiol-1,4-disubstituted-1,2,3-triazoles were obtained. Removal of the solvent under vacuum, followed by recrystallization with EtOH/H_2_O (1 : 1) afforded the pure β-thiol-1,4-disubstituted-1,2,3-triazoles derivatives in 80–96% yield (Table 2). All products are new compounds and were characterized by HRMS (EI), FT-IR, ^1^H NMR and ^13^C NMR spectra. The spectra of the products are given in the ESI.†

#### 2.5.1. Characterization of β-thiol-1,4-disubstituted-1,2,3-triazoles

##### 2.5.1.1. 2-Phenyl-2-(4-phenyl-1*H*-1,2,3-triazol-1-yl)-ethane-1-thiol (1b)


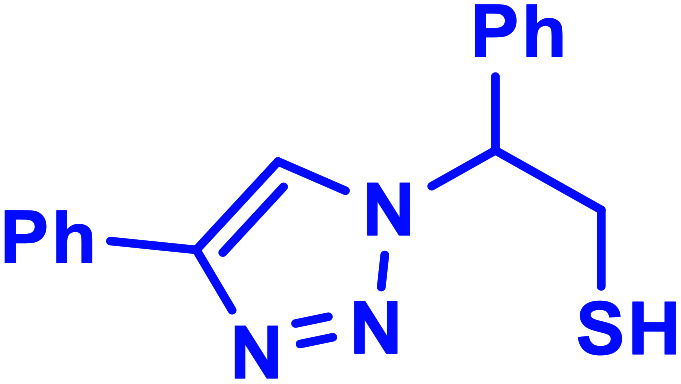
White solid: mp 122–124 °C, FT-IR (KBr): *ν*/cm^−1^ 3371, 3085, 3026, 2927, 2365, 1584, 1450, 1270, 1029, 762, 695; ^1^H NMR (500 MHz, CDCl_3_) *δ* 7.78–7.71 (m, 2H, Ar-H), 7.44–7.23 (m, 9H, Ar-H) 5.69 (dd, *J* = 7, 4.5 Hz, 1H, CHN), 4.63 (dd, *J* = 12.5, 5 Hz, 1H, CH_2_), 4.23 (dd, *J* = 15, 5 Hz, 1H, CH_2_), 3.63 (bs, 1H, SH); ^13^C NMR (125 MHz, CDCl_3_) *δ* 146.7 (=CN), 135.0 (NCH=), 128.1, 127.8, 127.2, 126.1, 124.7, 119.5 (10 × ArC), 66.2 (CHCH_2_), 28.6 (CH_2_). HRMS (EI) *m*/*z* calcd for C_16_H_15_N_3_S 281.0987, found 281.0985.

##### 2.5.1.2. 1-Phenoxy-3-(4-phenyl-1*H*-1,2,3-triazol-1-yl)propane-2-thiol (2b)


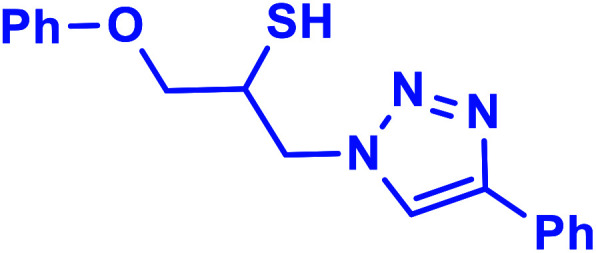
Milky solid: mp 113–114 °C, FT-IR (KBr): *ν*/cm^−1^ 3302, 3086, 2919, 2873, 2365, 1587, 1638, 1599, 1497, 1474, 1466, 1303, 1044, 764, 752, 712, 691; ^1^H NMR (500 MHz, CDCl_3_) *δ* 7.86 (s, 1H, NCH=C), 7.67–7.65 (m, 2H, Ar-H_o_), 7.35–7.31 (m, 2H, OAr-H_m_), 7.29–7.26 (m, 3H, Ar-H_m,p_), 7.00–6.87 (m, 3H, OAr-H_o,p_), 4.96 (bs, 1H, SH), 4.67 (dd, *J* = 13, 5 Hz, 1H, CH_2_O), 4.52 (dd, *J* = 13, 5 Hz, 1H, CH_2_O), 4.47 (dd, 1H, *J* = 12.5, 5 Hz, CH_2_N), 4.08 (dd, 1H, *J* = 12.5, 5 Hz, CH_2_N), 4.05–3.72 (m, 1H, CHS); ^13^C NMR (125 MHz, CDCl_3_) *δ* 157.1 (=CN), 146.53, 129.1 (2 × ArC), 128.6, 128.5, 127.8, 127.1, 124.6, 120.5, 120.4 (10 × ArCH), 113.5 (NCH=), 67.8 (CH_2_O), 52.2 (CH_2_N), 28.6 (CHS). HRMS (EI) *m*/*z* calcd for C_17_H_17_N_3_OS 311.1092, found 311.1092.

##### 2.5.1.3. 1-Chloro-3-(5-phenyl-1*H*-1,2,3-triazol-1-yl)propane-2-thiol (3b)


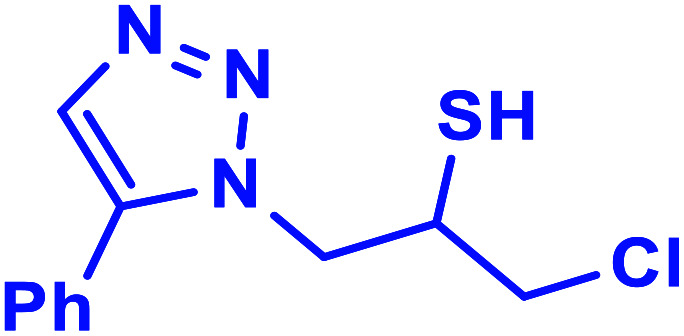
Mint white solid: mp 154–157 °C FT-IR (KBr): *ν*/cm^−1^ 3690, 3100, 2920, 2363, 2344, 1561, 1441, 1364, 1045, 764, 692, 668. ^1^H NMR (500 MHz, CDCl_3_) *δ* 7.93 (s, 1H, NCH=C), 7.88–7.23 (m, 5H, Ar-H), 4.61–4.41 (m, 2H, CH_2_N), 4.39–4.31 (m, 1H, CH_2_Cl), 4.00–3.94 (m, 1H, CH_2_Cl), 3.90–3.37 (m, 1H, CHS), 2.07 (bs, 1H, SH); ^13^C NMR (125 MHz, CDCl_3_) *δ* 148.0 (=CN), 130.1 (ArC, NCH=), 128.9, 128.4, 125.7, 117.6 (5 × ArCH), 55.9 (CH_2_N), 34.4 (CH_2_Cl), 29.6 (CHS). HRMS (EI) *m*/*z* calcd for C_11_H_12_ClN_3_S 253.0441, found 253.0442.

##### 2.5.1.4. 1-(4-Phenyl-1*H*-1,2,3-triazol-1-yl)butane-2-thiol (4b)


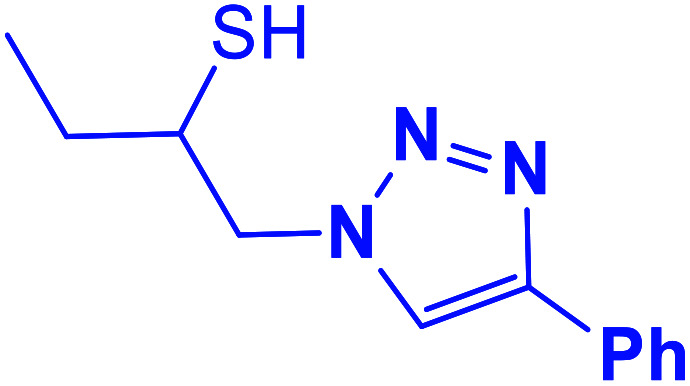
White solid: mp 104–106 °C, FT-IR (KBr): *ν*/cm^−1^ 3692, 3139, 2958, 2873, 2366, 2345, 1585, 1439, 1230, 1072, 765, 694; ^1^H NMR (500 MHz, CDCl_3_) *δ* 7.83 (s, 1H, NCH=C), 7.68–7.23 (m, 5H, Ar-H), 4.49 (dd, *J* = 14, 5 Hz, 1H, CH_2_N), 4.24 (dd, *J* = 14, 8 Hz, 1H, CH_2_N), 4.09–4.04 (m, 1H, CHS), 3.43 (bs, 1H, SH), 1.61–1.55 (m, 2H, CH_2_), 1.06 (t, *J* = 7.5 Hz, 3H, CH_3_); ^13^C NMR (125 MHz, CDCl_3_) *δ* 147.3 (=CN), 130.3 (NCH=), 128.7, 128.0, 125.5, 121.1 (6 × ArC), 71.7 (CH_2_N), 55.9 (CHS), 27.4 (CH_2_), 9.8 (CH_3_). HRMS (EI) *m*/*z* calcd for C_12_H_15_N_3_S 233.0987, found 233.0986.

##### 2.5.1.5. 1-Isopropoxy-3-(4-phenyl-1*H*-1,2,3-triazol-1-yl)propane-2-thiol (5b)


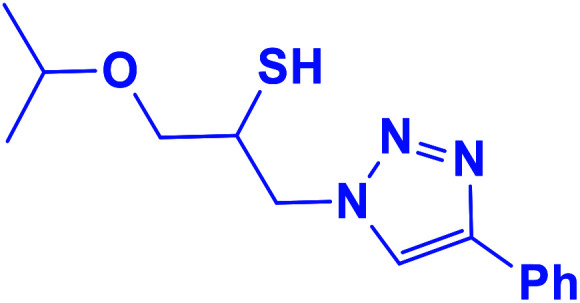
Yellow solid: mp 74–75 °C, FT-IR (KBr): *ν*/cm^−1^ 3177, 2968, 2875, 2369, 2345, 1583, 1460, 1444, 1366, 1071, 767, 702; ^1^H NMR (500 MHz, CDCl_3_) *δ* 7.86 (s, 1H, NCH=C), 7.67–7.65 (m, 2H, Ar-H), 7.33–7.23 (m, 3H, Ar-H), 4.53 (dd, *J* = 14, 4 Hz, 1H, CH_2_N), 4.34 (dd, *J* = 14, 7.5 Hz, 1H, CH_2_N), 4.21–4.16 (m, 2H, OCH_2_), 3.58–3.51 (m, 1H, CHO), 3.46–3.35 (m, 2H, CHS overlapped with SH), 1.11 (d, *J* = 5 Hz, 6H, 2CH_3_); ^13^C NMR (125 MHz, CDCl_3_) *δ* 147.9 (=CN), 130.3 (NCH=), 128.7, 128.0, 125.5, 121.4 (6 × ArC), 72.3 (CHO), 69.2 (OCH_2_), 69.1 (CH_2_N), 53.4 (CHS), 21.9 (2 × CH_3_). HRMS (EI) *m*/*z* calcd for C_14_H_19_N_3_OS 277.1249, found 277.1247.

##### 2.5.1.6. 2-(4-Phenyl-1*H*-1,2,3-triazol-1-yl)cyclohexane-1-thiol (6b)


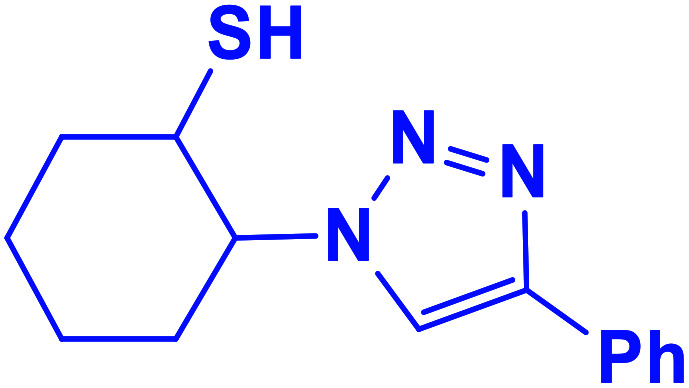
Pale green solid: mp 156–159 °C, FT-IR (KBr): *ν*/cm^−1^ 3305, 3094, 2936, 2853, 2364, 1586, 1440, 1236, 1055, 766, 698; ^1^H NMR (500 MHz, CDCl_3_) *δ* 7.83 (s, 1H, NCH=C), 7.63–7.25 (m, 5H, Ar-H), 4.12–4.04 (m, 1H, CHN), 3.57–2.94 (m, 1H, CHS), 2.04–1.22 (m, 4 × CH_2_ overlapped with 1H, SH); ^13^C NMR (125 MHz, CDCl_3_) *δ* 146.6 (=CN), 132.4 (NCH=), 130.2, 128.6, 127.9, 125.4, 119.8 (6 × ArC), 72.5 (CHN), 67.2 (CHS), 28.6 (CHS), 33.8, 31.5, 24.8, 24.0 (4 × CH_2_). HRMS (EI) *m*/*z* calcd for C_14_H_17_N_3_S 259.1143, found 259.1143.

##### 2.5.1.7. 1-(Allyloxy)-3-(4-phenyl-1*H*-1,2,3-triazol-1-yl)propane-2-thiol (7b)


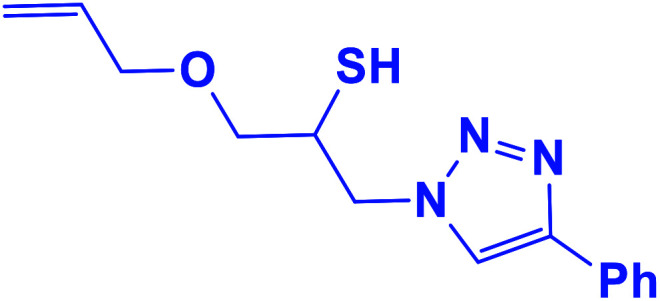
Cream solid: mp 70 °C, FT-IR (KBr): *ν*/cm^−1^ 3233, 3141, 2930, 2872, 2366, 1590, 1438, 1350, 1230, 1000, 767, 696; ^1^H NMR (500 MHz, CDCl_3_) *δ* 7.85 (s, 1H, NCH=C), 7.55–7.23 (m, 5H, Ar-H), 5.80–5.87 (m, 1H, =CH), 5.22 (dt, *J* = 17, 1.2 Hz, 1H, C=CH_2_), 5.14 (dt, *J* = 10, 1.2 Hz, 1H, C=CH_2_), 4.55–4.50 (m, 2H, CH_2_O), 4.35 (dd, *J* = 15, 7 Hz, 1H, CH_2_N), 4.26–4.23 (m, 1H, CH_2_N), 4.00–3.88 (m, 2H, OCH_2_), 348–3.41 (m, 1H, CHS), 2.91 (bs, 1H, SH); ^13^C NMR (125 MHz, CDCl_3_) *δ* 147.1 (=CN), 134.2 (NCH=), 130.2 (=CH), 128.7, 128.0, 125.5, 121.5 (6 × ArC), 117.4 (=CH_2_), 72.3 (OCH_2_), 71.2 (CH_2_O), 69.0 (CH_2_N), 53.4 (CHS). HRMS (EI) *m*/*z* calcd for C_14_H_17_N_3_OS 275.1092, found 275.1094.

##### 2.5.1.8. 2-Mercapto-3-(4-phenyl-1*H*-1,2,3-triazol-1-yl)propyl methacrylate (8b)


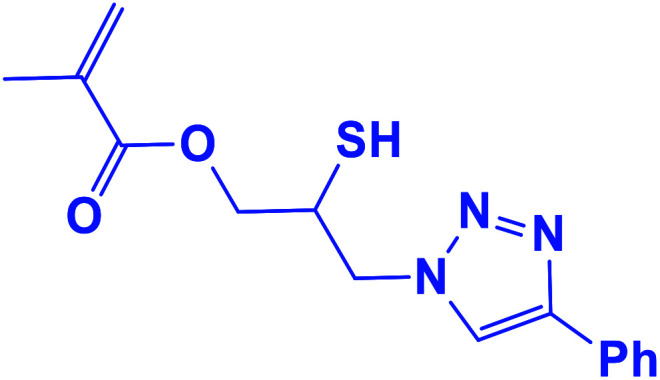
Milky solid: mp 86–92 °C, FT-IR (KBr): *ν*/cm^−1^ 3000, 2925, 2367, 1719, 1593, 1438, 1294, 1097, 762, 695; ^1^H NMR (300 MHz, CDCl_3_): *δ* 7.86 (s, 1H, NCH=C), 7.66–7.64 (m, 2H, Ar-H), 7.34–7.25 (m, 3H, Ar-H), 6.10 (d, *J* = 10 Hz, 1H, =CH_2_), 5.93 (d, *J* = 10 Hz, 1H, =CH_2_), 4.48–4.12 (m, 2H, OCH_2_), 3.57 (m, 2H, CH_2_N), 3.66 (bs, 1H, SH), 3.33–3.32 (m, 1H, CSH), 2.05 (s, 3H, CH_3_); ^13^C NMR (125 MHz, CDCl_3_): *δ* = 161.6 (C=O), 147.3 (=CN), 135.7 (C=), 128.9 (NCH=), 128.8, 128.2, 125.5, 121.6 (6 × ArC), 70.6 (OCH_2_), 63.6 (CH_2_N), 53.0 (CHS), 18.3 (CH_3_). HRMS (EI) *m*/*z* calcd for C_15_H_17_N_3_O_2_S 303.1042, found 303.1045.

##### 2.5.1.9. 2-(4-Cyclohexyl-1*H*-1,2,3-triazol-1-yl)-2-phenylethane-1-thiol (9b)


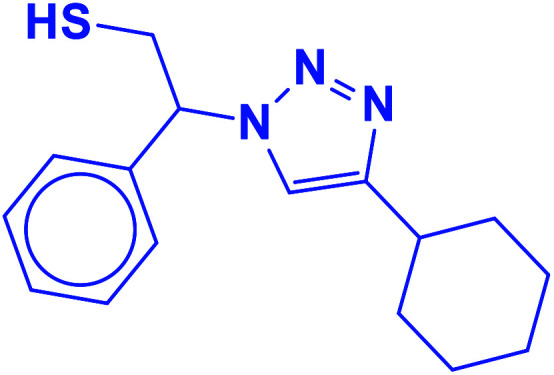
White solid: mp 125–128 °C, FT-IR (KBr): *ν*/cm^−1^ 3233, 3117, 3061, 2923, 2849, 1448, 1067, 889, 756, 697; ^1^H NMR (500 MHz, CDCl_3_): *δ* 7.60 (s, 1H, NCH=C), 7.42–7.26 (m, 5H, Ar-H), 5.68 (dd, *J* = 8.7, 4.8 Hz, 1H, NCHCH_2_), 4.40–4.28, 4.15–4.03 (2 m, 2H, CH_2_S), 3.32 (t, *J* = 5.9 Hz, 1H, SH), 2.76–2.68 (m, 1H, CH_2_CHCH_2_), 1.76–1.65 (m, 4H, 2 × CH_2_CH_2_CH), 1.43–1.36 (m, 4H, 2 × CH_2_CH_2_CH), 1.27–1.18 [m, 2H, (CH_2_)_2_CH_2_(CH_2_)_2_]. ^13^C NMR (125 MHz, CDCl_3_): *δ* = 153.9 (NCCH), 138.5 (ArC), 129.7, 129.3, 128.1 (5 × ArCH), 120.9 (NCCH), 64.7 (NCHCH_2_), 53.3 (CH_2_S), 36.1 (CH_2_CHCH_2_), 33.8, 26.9, 26.8 [(CH_2_)_5_]; HRMS (EI) *m*/*z* calcd for C_16_H_21_N_3_S 287.1456, found 287.1455.

##### 2.5.1.10. (1-(2-Mercapto-1-phenylethyl)-1*H*-1,2,3-triazol-4-yl)methanol (10b)


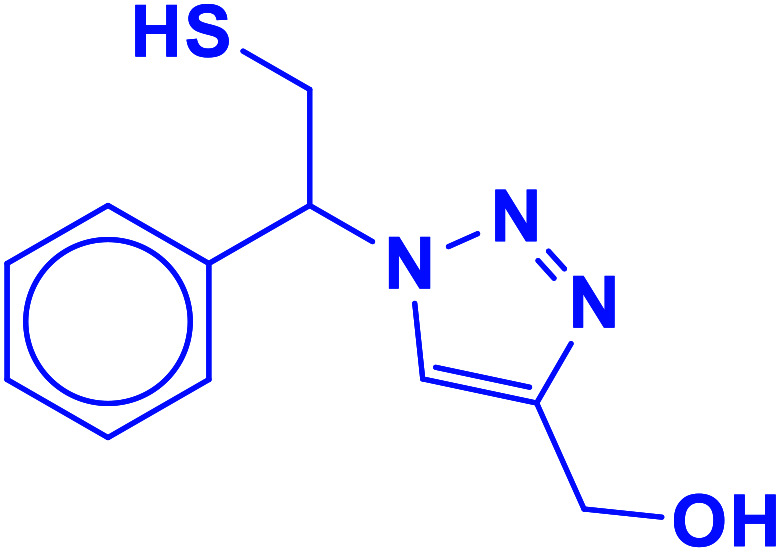
Colorless solid: mp 101–105 °C, FT-IR (KBr): *ν*/cm^−1^ 3340, 3167, 2932, 2893, 1454, 1234, 1119, 1084, 1011, 849, 795, 702. ^1^H NMR (500 MHz, CDCl_3_): *δ* 7.62 (s, 1H, NCH=C), 7.37–7.28 (m, 5H, Ar-H), 5.68 (dd, *J* = 8.7, 4.8 Hz, 1H, NCHCH_2_), 4.52 (s, 2H, CH_2_O), 4.38–4.30, 4.13–4.02 (2 m, 2H, CH_2_S), 3.76 (bs, 1H, OH), 3.32 (t, *J* = 5.9 Hz, 1H, SH), ^13^C NMR (125 MHz, CDCl_3_): *δ* = 147.1 (=CN), 130.1 (ArC), 128.5, 128.0, 125.7 (5 × ArCH), 121.3 (=CHN), 71.8 (CHN), 55.7 (CH_2_O), 27.3 (CH_2_S). HRMS (EI) *m*/*z* calcd for C_11_H_13_N_3_OS 235.0779, found 235.0781.

##### 2.5.1.11. 2-(4-(4-Methoxyphenyl)-1*H*-1,2,3-triazol-1-yl)-2-phenylethane-1-thiol (11b)


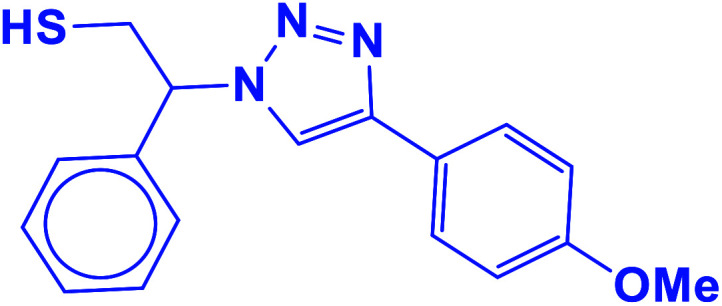
White solid: mp 130–132 °C, FT-IR (KBr): *ν*/cm^−1^ 3371, 3085, 3026, 2927, 2365, 1584, 1450, 1270, 1074, 1041, 1029, 762, 695; ^1^H NMR (500 MHz, CDCl_3_) *δ* 7.76–7.71 (m, 3H, Ar-H), 7.44–7.21 (m, 7H, Ar-H) 5.71 (dd, *J* = 7, 4.5 Hz, 1H, CHN), 4.15 (s, 3H, OCH_3_), 3.76 (dd, *J* = 12.5, 5 Hz, 1H, CH_2_), 3.37 (dd, *J* = 15, 5 Hz, 1H, CH_2_), 2.75 (bs, 1H, SH); ^13^C NMR (125 MHz, CDCl_3_) *δ* 146.6 (=CN), 135.0 (NCH=), 128.1, 127.7, 127.2, 126.1, 124.7, 119.1 (10 × ArC), 66.2 (CHN), 58.26 (OCH_3_), 28.6 (CH_2_S). HRMS (EI) *m*/*z* calcd for C_17_H_17_N_3_OS 311.1092, found 311.1095.

### 2.6. Recycling of MgFe_2_O_4_/Cu nanocatalyst

The MgFe_2_O_4_/Cu nanoparticles were recovered with an external magnet, washed several times with ethyl acetate and distilled water, and used for the subsequent cycles after drying under air atmosphere.

## 3. Results and discussion

### 3.1. Synthesis and characterization of MgFe_2_O_4_/Cu nanocatalyst

Although, MgFe_2_O_4_ has a large surface to volume ratio and therefore possesses high catalytic capability due to its wide contact surface, it tends to aggregate so as to minimize the surface energies. Moreover, the naked magnesium ferrite nanoparticles have high chemical activity, and are easily oxidized in air, generally resulting in loss of magnetic property and dispersibility. Therefore, it is significant to provide appropriate surface coating to keep the stability of MgFe_2_O_4_ particles. Coating with an inorganic layer, such as silica, metal or nonmetal elementary substance and metal oxide is important because the protecting shells not only reduces the aggregation of the nanoparticles in the solution and stabilize the magnetic nano-ferrite, but can also be used for further functionalization and improves the efficiency of the catalyst.^56^ Ferrites are highly valuable catalyst supports because they take advantage of magnetic property and can provide unique features such as easy separation, recoverability and reusability for the new synthesized MgFe_2_O_4_/Cu nanocatalyst.

**Scheme 2:**
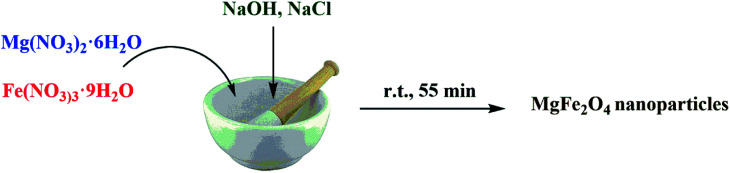
Synthesis of MgFe_2_O_4_ nanoparticles.

**Scheme 3:**
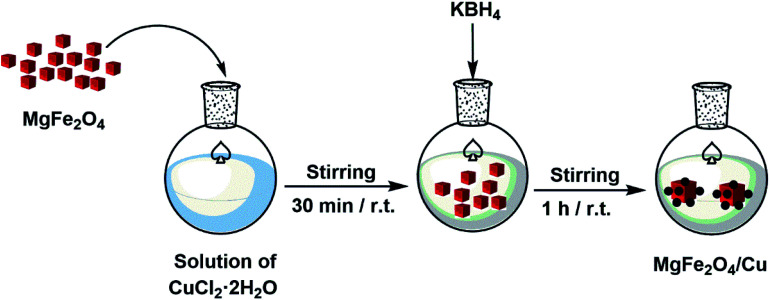
Synthesis of MgFe_2_O_4_/Cu nanocomposite.

The nanoparticles of MgFe_2_O_4_/Cu were synthesized in a two-step procedure. MgFe_2_O_4_ was prepared using solid-state reaction of Mg(NO_3_)_2_·6H_2_O, Fe(NO_3_)_3_·9H_2_O, NaOH, and NaCl in a mortar (Scheme 2). After calcination of crude powder at 900 °C, the MgFe_2_O_4_ nanoparticles were obtained with high crystallinity and phase purity. Then, MgFe_2_O_4_ nanoparticles were added to an aqueous solution of CuCl_2_·2H_2_O and followed by gradually addition of KBH_4_ powder under intense stirring. Finally, the black precipitate was collected through magnetic separation, washed with deionized water, and dried at room temperature (Scheme 3). The prepared MgFe_2_O_4_/Cu nanocatalyst was characterized by various techniques such as FT-IR, vibration sample magnetometer (VSM), X-ray diffraction (XRD), transmission electron microscopy (TEM), field emission scanning electron microscope (FESEM), energy dispersive X-ray spectrometer (EDS), and inductively coupled plasma optical emission spectrometry (ICP-OES) analyses.

#### 3.1.1. Vibration sample magnetometer (VSM)

**Fig. 1:**
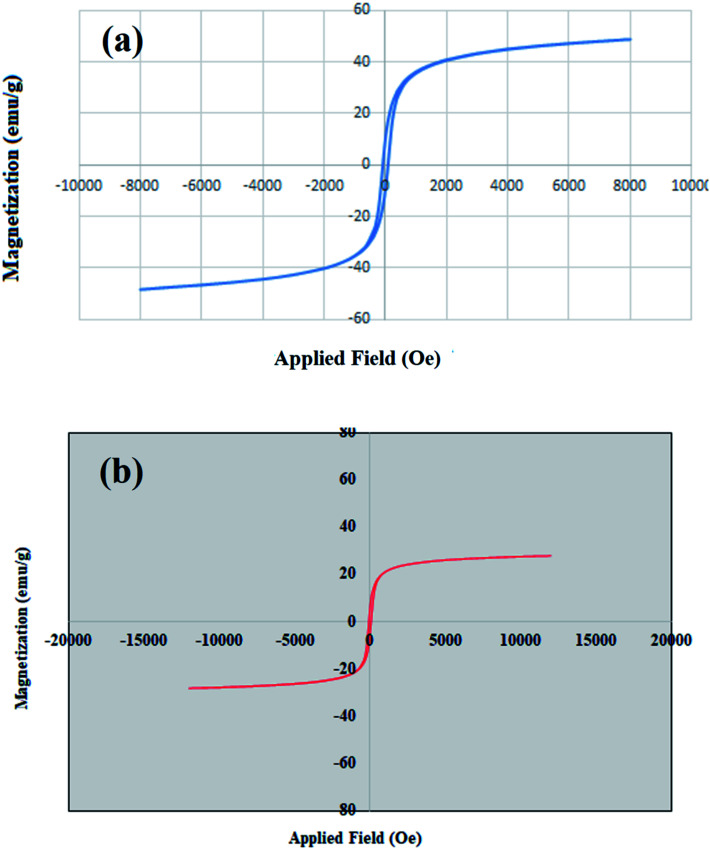
Magnetization curves of (a) MgFe_2_O_4_ (b) MgFe_2_O_4_/Cu nanoparticles.

The hysteresis loops, saturation magnetization (Ms) and switching field (Hc) of MgFe_2_O_4_ and MgFe_2_O_4_/Cu nanoparticles are shown in Fig. 1. MgFe_2_O_4_/Cu nanoparticles show lower magnetization saturation (27 emu g^−1^) than the uncoated magnesium ferrite nanoparticles (48 emu g^−1^). This is owing to the effect of copper shell coating where each ferrite particle was separated from its neighbors by the coated layer leading to diminish the magnetostatic coupling between the particles. The samples exhibit typical ferromagnetic behavior at room temperature. The narrow cycles and the hysteresis loops show the behavior of soft magnetic materials with low coercivity.

#### 3.1.2. Fourier transform infra-red (FT-IR) spectrum

**Fig. 2:**
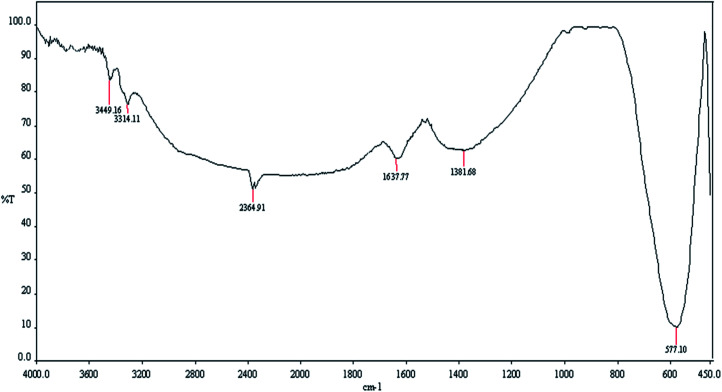
FT-IR (KBr) spectrum of MgFe_2_O_4_/Cu nanoparticles.

Fig. 2 shows the FT-IR spectrum of MgFe_2_O_4_/Cu nanocatalyst. The absorption band at 577 cm^−1^ is related to the vibration of metal oxide bonds (Fe–O and Mg–O) which confirms the formation of MgFe_2_O_4_ nanoparticles. The absorption peaks at 3314 and 3449 cm^−1^ are assigned to the stretching vibration of H_2_O molecules and indicates the OH groups on the surface of the magnetic nanoparticles. The band around 1637 cm^−1^ corresponds to the bending mode of H_2_O molecules.^51^

#### 3.1.3. X-ray diffraction (XRD)

**Fig. 3:**
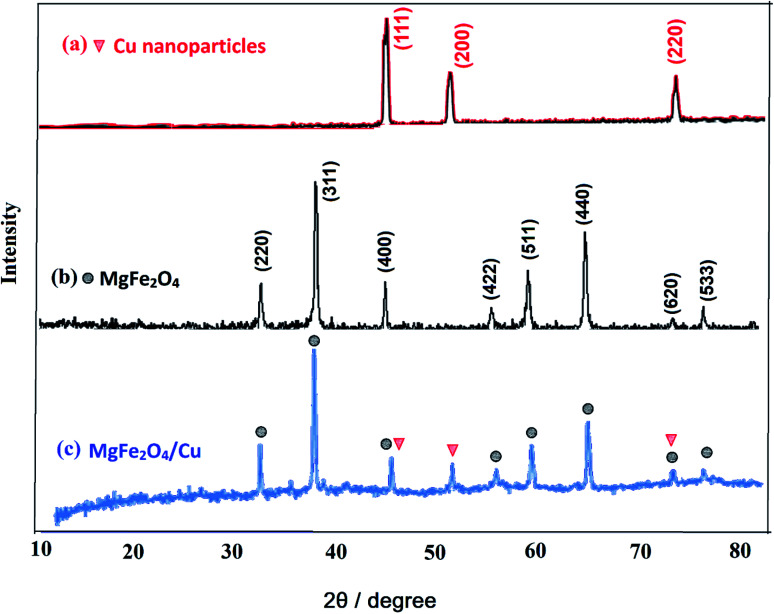
The X-ray diffraction patterns of (a) nano-Cu, (b) nano-MgFe_2_O_4_ and (c) MgFe_2_O_4_/Cu nanocomposite.

Fig. 3 shows the X-ray diffraction (XRD) patterns of MgFe_2_O_4_/Cu, MgFe_2_O_4_ and copper nanoparticles. In the XRD pattern of MgFe_2_O_4_/Cu, all the peaks of MgFe_2_O_4_ and Cu nanoparticles are traceable. The lines (220), (311), (400), (422), (511), (440), (620) and (533) related to 2*θ* = 30.22°, 35.60°, 37.18°, 43.27°, 57.09°, 62.68°, 74.38° and 75.39° respectively, are assigned to the diffraction of MgFe_2_O_4_ crystals and indicate that the synthesized MgFe_2_O_4_ nanoparticles are pure and high crystalline. These peaks are compatible with the standard data (JCPDS: 01-036-0398).^51^ The characteristic diffraction peaks of copper located at 2*θ* = 43.7°, 50.7°, and 74.3° which correspond to the (111), (200), and (220) planes of the fcc structure, respectively and they are in good agreement with copper standard (JCPDS: 04-0836).^57^ The peaks at 43.7° and 74.3° for copper overlap with the 43.27° and 74.38° peaks of MgFe_2_O_4_, respectively. The average crystallite size of MgFe_2_O_4_/Cu nanoparticles is calculated using the Scherrer's formula (46 nm).

#### 3.1.4. TEM, FESEM and EDS of MgFe_2_O_4_/Cu

**Fig. 4:**
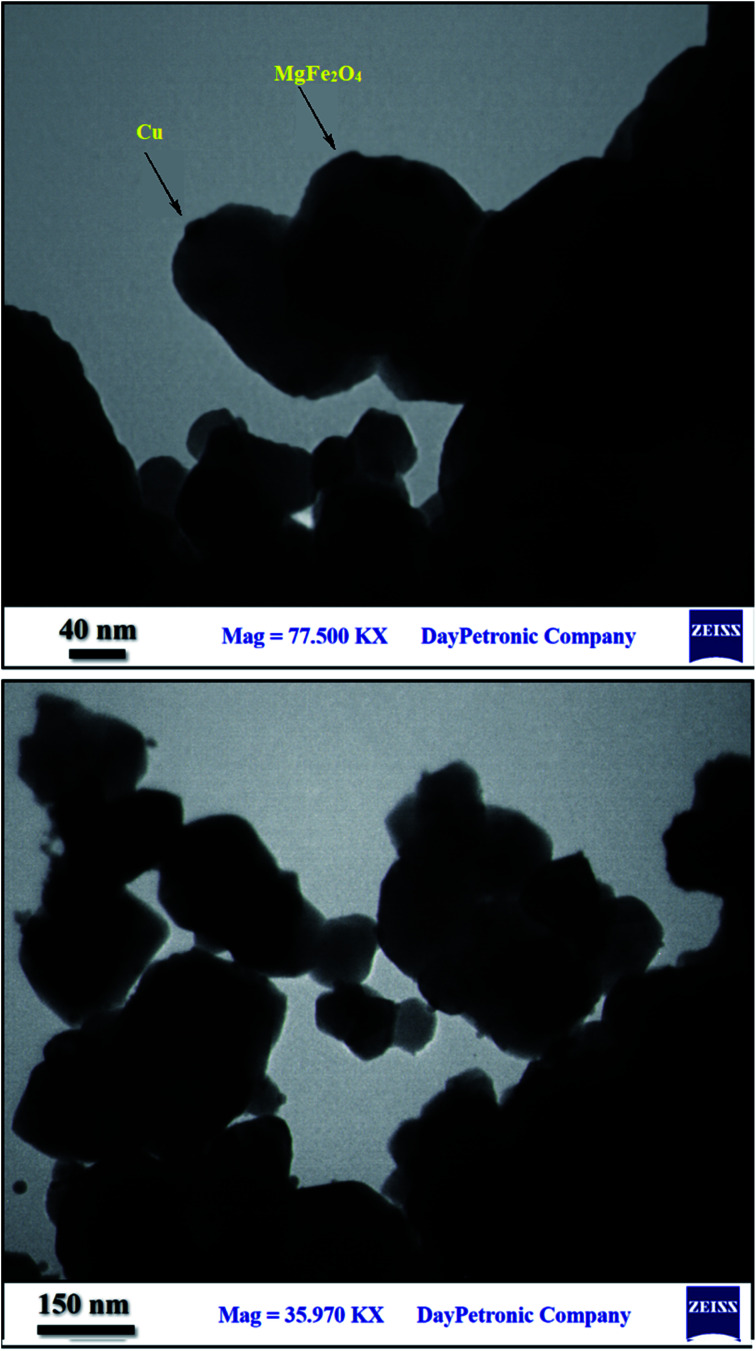
TEM images of MgFe_2_O_4_/Cu.

The morphology and size distribution of the synthesized nanocatalyst have been studied by TEM and FESEM techniques. TEM images of the MgFe_2_O_4_/Cu nanocomposite are shown in Fig. 4. As can be seen from the images, two sizes of particles are clearly distinguishable, with differences in their colour and morphology. The larger grey spots with cubic shape were attributed to the MgFe_2_O_4_ particles which coated with the small black segments of copper nanoparticles.

**Fig. 5:**
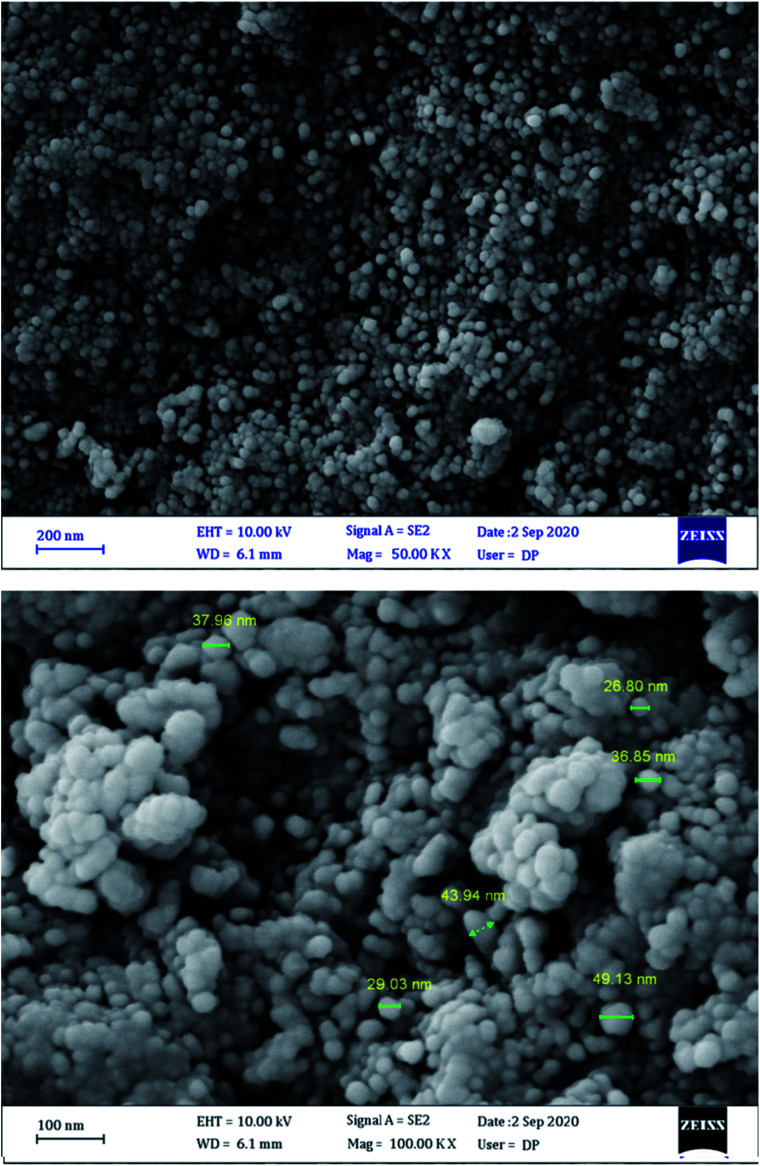
FESEM images of MgFe_2_O_4_/Cu.

Fig. 5 shows FESEM images of MgFe_2_O_4_/Cu nanocomposite that confirm the presence of nanoparticles with diameters ranging from 29 to 43 nm. The obtained results are in good agreement with TEM and XRD data.

**Fig. 6:**
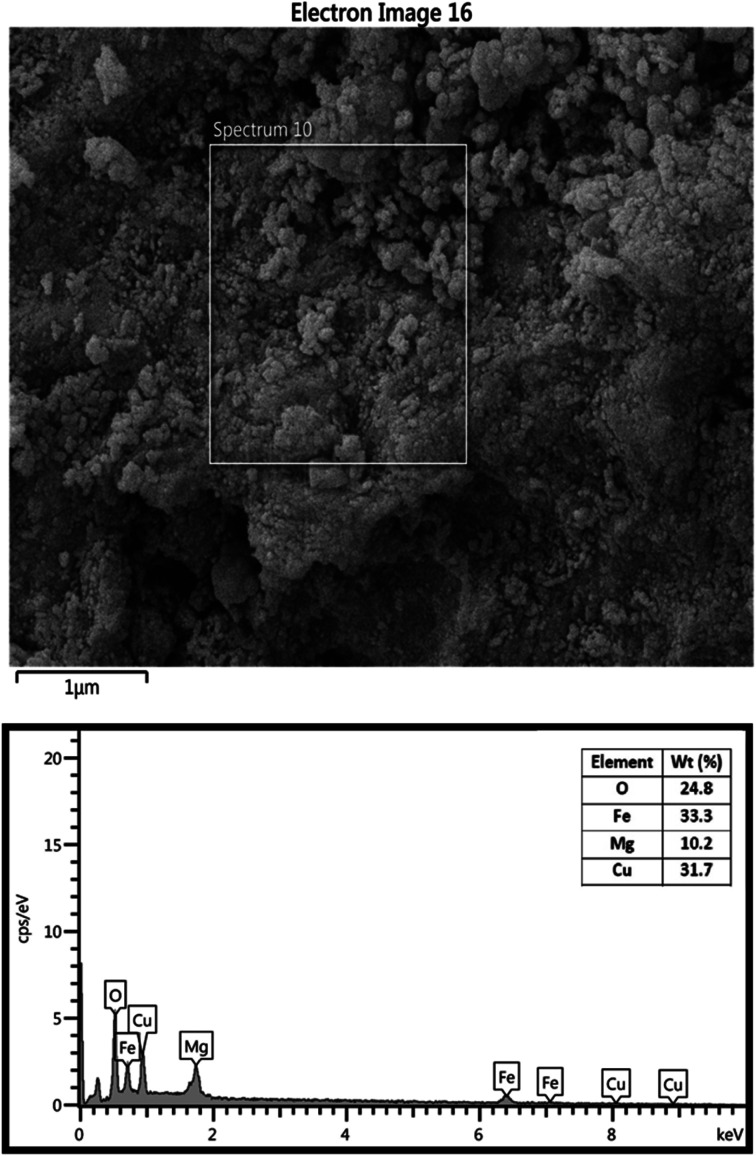
EDS of MgFe_2_O_4_/Cu.

The chemical composition of MgFe_2_O_4_/Cu nanocomposite was confirmed with EDS data. In this analysis, Cu, Mg, Fe, and O signals are observable (Fig. 6). Additionally, the exact concentration of Mg, Fe and Cu was determined by ICP-OES and the obtained values were 10.2, 33.35 and 31.68 wt% respectively, which are in good agreement with EDS data.

### 3.2. Catalytic activity of MgFe_2_O_4_/Cu for the synthesis of β-thiol-1,4-disubstituted-1,2,3-triazoles

In order to optimize the reaction conditions, we investigated the one-pot click synthesis of 2-phenyl-2-(4-phenyl-1*H*-1,2,3-triazol-1-yl)ethane-1-thiol from styrene episulfide, sodium azide and phenyl acetylene under various reaction conditions. Initially, temperature, solvents, reaction time and the amounts of catalyst and reactants were studied as experimental factors, and then the results were summarized in Table 1. The favorable outcome was obtained using styrene episulfide (1 mmol), sodium azide (1.2 mmol) and phenylacetylene (1 mmol) in the presence of nano-MgFe_2_O_4_/Cu (0.02 g) as catalyst in water at 60 °C (Table 1, entry 4). It is noteworthy that the presence of catalyst was necessary to perform the reaction and in the absence of nanocomposite, the reaction did not proceed even after 11 h (entry 1). The quantity of catalyst was optimized using various amounts of nano-MgFe_2_O_4_/Cu (0.005, 0.01, 0.02 and 0.03 g), and the best result was obtained with 0.02 g of catalyst. The catalyst concentration plays a significant role in the optimization of the product yield. An increase in the amount of catalyst from 0.01 to 0.02 g not only increased the triazole yield but also accelerated the rate of reaction (entries 2–4). Using the more amounts of nanocatalyst did not improve the product yield (entry 5).

**Table 1:** Nano-MgFe_2_O_4_/Cu-catalysed reaction of styrene episulfide with phenylacetylene and sodium azide under different conditions^a^

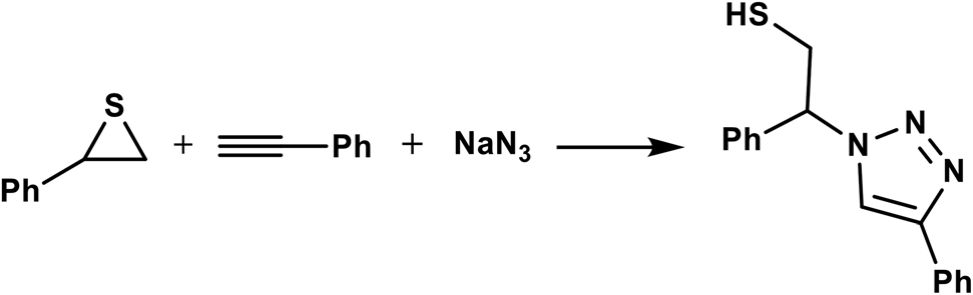					
Entry	MgFe_2_O_4_/Cu (g)	Solvent	Time (h)	Temperature (°C)	Yield^b^ (%)
1	—	H_2_O	11	60	0
2	0.005	H_2_O	5	60	40
3	0.01	H_2_O	5	60	75
**4**	**0.02**	**H**_**2**_ **O**	**2.5**	**60**	**96**
5	0.03	H_2_O	2.4	60	96
6	0.02	CH_3_CN	3	82	50
7	0.02	EtOH	3	78	60
8	0.02	MeOH	3	65	60
9	0.02	EtOAc	3	77	65
10	0.02	DMF	3	100	55
11	0.02	THF	20	60	0
12	0.02	*n*-Hexane	20	68	0
13	0.02	CCl_4_	20	77	0
14	0.02	H_2_O	10	25	45
15	0.02	H_2_O	6	45	70
16^c^	0.02	H_2_O	4	60	Trace
17^d^	0.02	H_2_O	3	60	92

a All reactions were carried out with styrene episulfide (1 mmol), phenylacetylene (1 mmol) and sodium azide (1.2 mmol).

b Isolated yields.

c Catalysed by MgFe_2_O_4_.

d Catalysed by Cu nanoparticles.

In order to study of solvent effect, the cyclization reaction was tested in the various solvents. The results showed that the polar solvents such as water, acetonitrile, ethanol, methanol, ethyl acetate and dimethylformamide were effective and utilizable whereas non-polar solvents were not suitable for this purpose (entries 6–13). The reaction was carried out successfully in H_2_O and it was selected as the best option because in comparison with water, the product yields were lower in all other solvents and also water is a green and eco-friendly solvent (entry 4).

The effect of temperature was also investigated and the reaction was tested at different temperatures (25, 45 and 60 °C). The product yield was not satisfactory at room temperature (25 °C) after 10 h (entry 14). Increasing the temperature simultaneously increased the reaction rate and product yield, and the desired triazole was synthesized in 70% yield after 6 h at 45 °C (entry 15). Further increase of temperature up to 60 °C led to produce the product with excellent yield at short reaction time (entry 4). The reaction was tested in the presence of bare MgFe_2_O_4_ and Cu nanoparticles separately under the optimized conditions and results showed that although magnesium ferrite nanoparticles improve and enhance the catalytic activity of nanocomposite, copper particles play an essential role for proceeding the reaction and their presence is vital in triazole cyclization (entries 16 and 17).

The generality of the presented procedure was established by reaction of various thiiranes bearing either electron-donating or withdrawing substituents, and cyclic thiiranes with phenylacetylene and sodium azide in the presence of MgFe_2_O_4_/Cu nanocomposite under the optimized conditions. The results are summarized in Table 2. In addition, the reaction of other alkynes such as aliphatic terminal alkynes and 4-methoxyphenyl acetylene with styrene episulfide was also considered under mentioned conditions (entries 9–11). All reactions were carried out successfully within 2–4 h to give triazoles in 80–96% yields.

**Table 2:** One-pot synthesis of β-Thiol-1,4-disubstituted-1,2,3-triazoles from thiiranes catalyzed by nano-MgFe_2_O_4_/Cu^a^

Entry	Thiirane (a)	Alkyne	Triazole (b)	Time (h)	Yield^b^ (%)
1	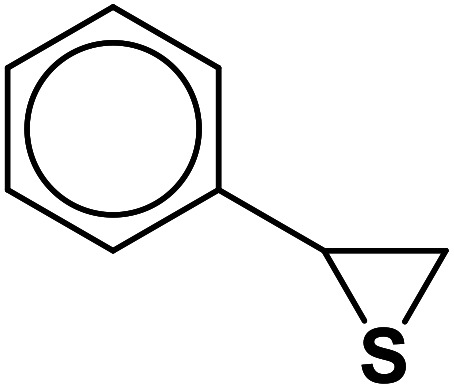	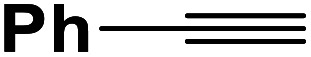	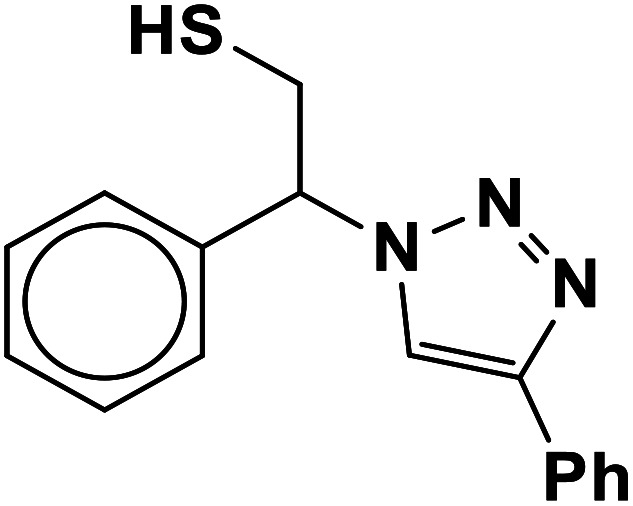	2.5	96
2	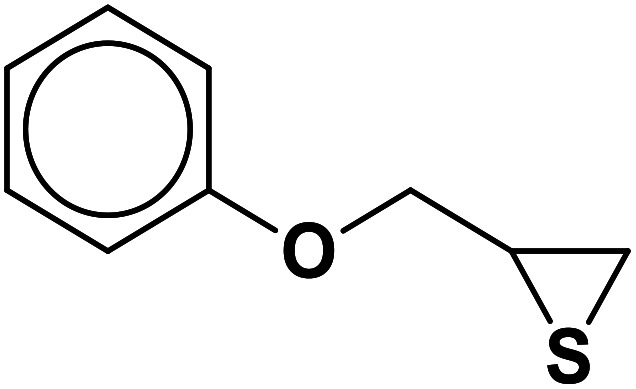	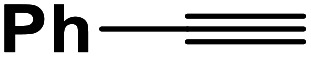	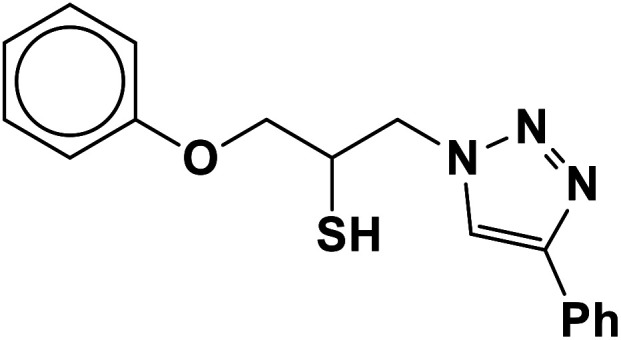	2	91
3	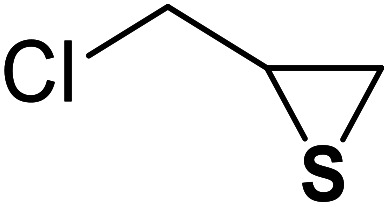	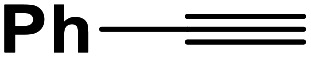	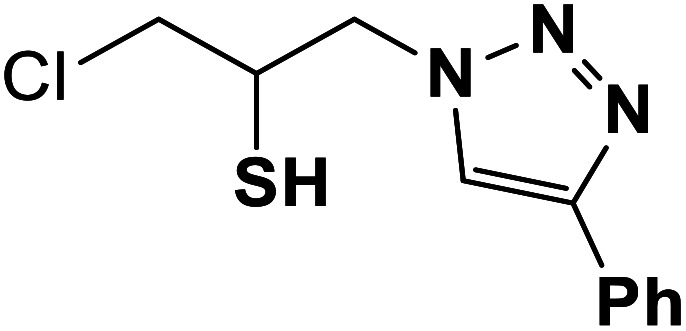	2.5	80
4	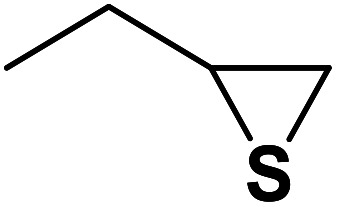	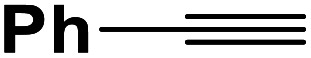	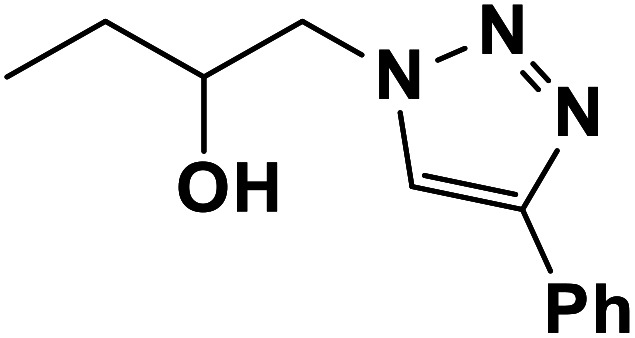	4	95
5	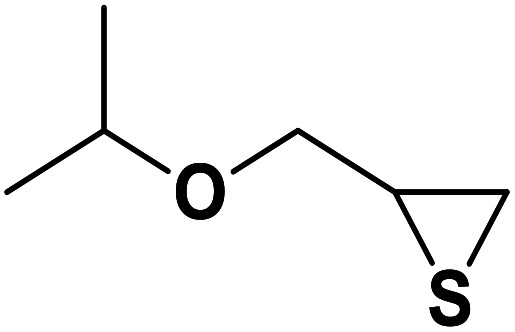	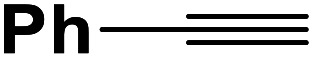	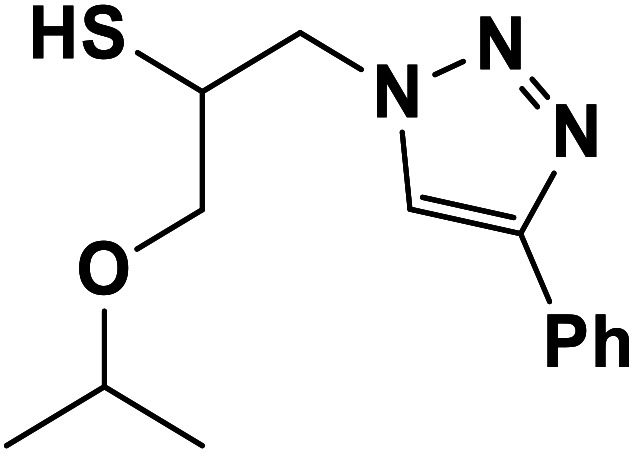	3	90
6	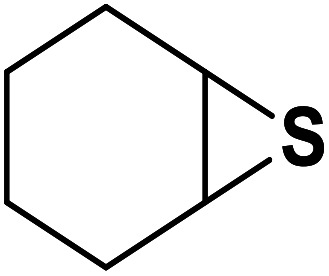	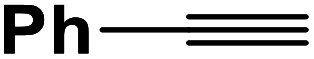	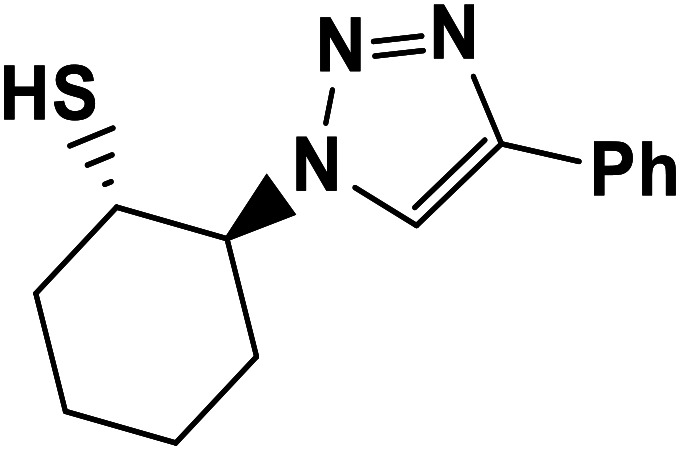	2	94
7	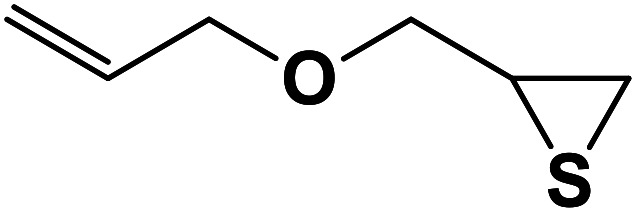	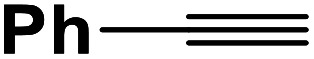	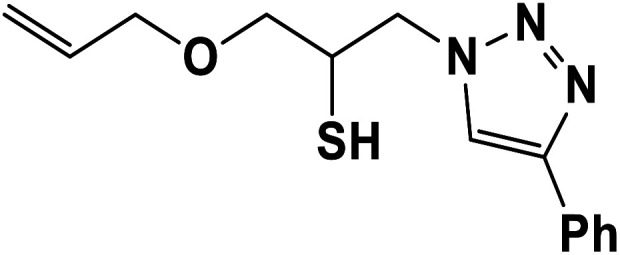	2.5	90
8	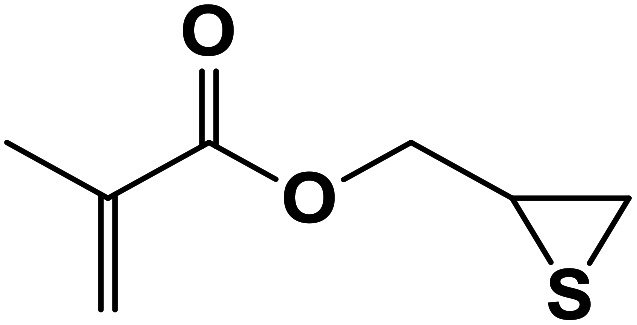	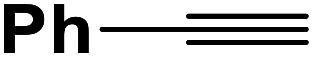	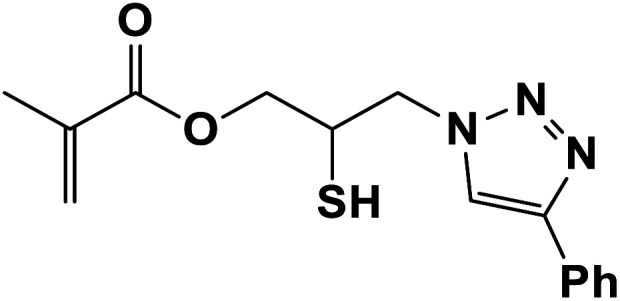	2	80
9	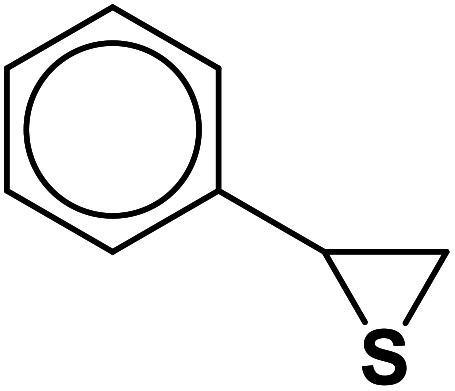	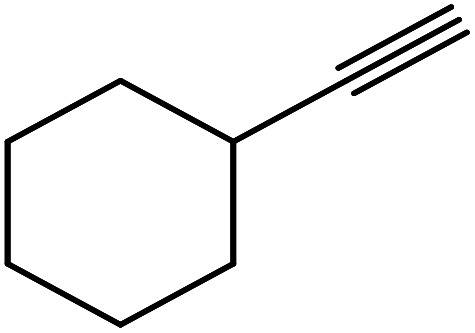	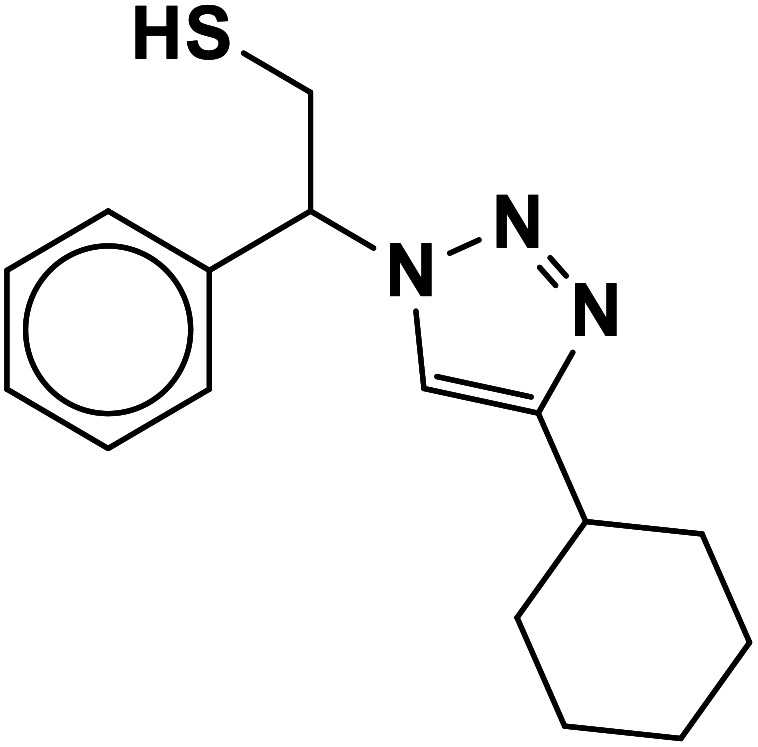	3	92
10	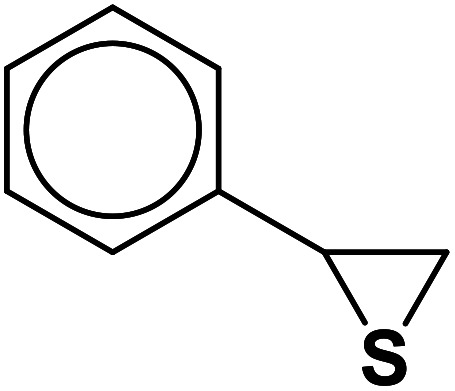	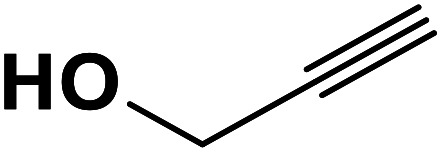	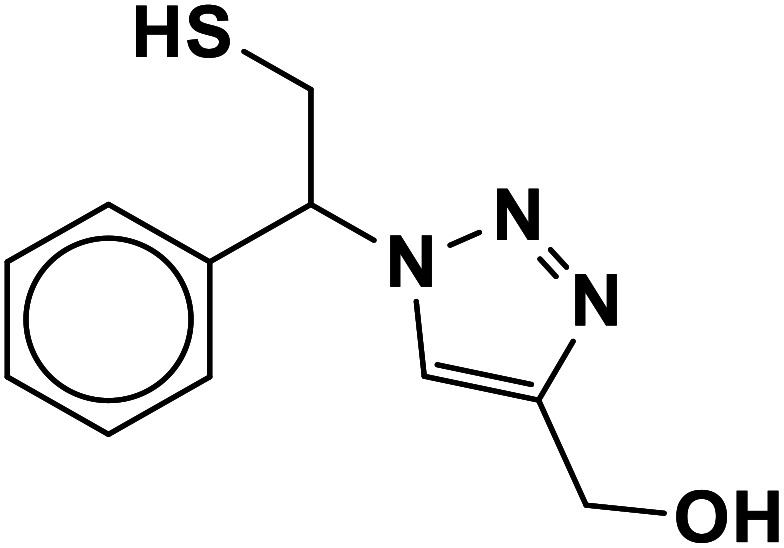	3.5	90
11	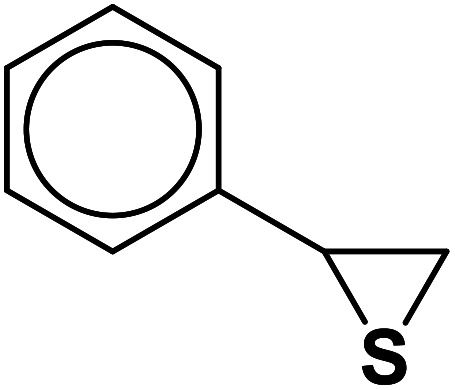	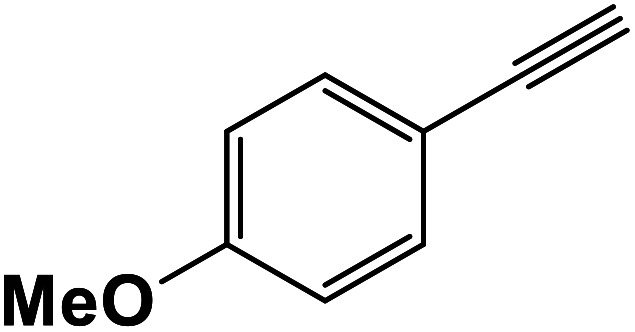	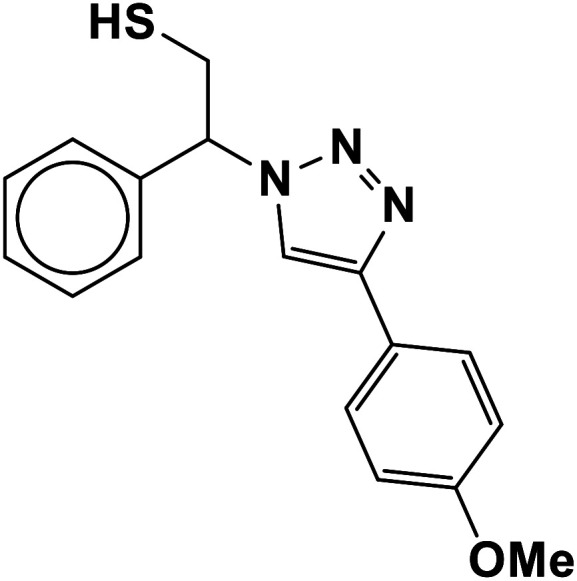	2.5	95

a All reactions were carried out with 1 mmol of thiirane in the presence of alkyne (1 mmol), sodium azide (1.2 mmol) and nano-MgFe_2_O_4_/Cu (0.02 g) in water at 60 °C.

b Yields refer to isolated pure products.

### 3.3. Recycling of nano-MgFe_2_O_4_/Cu

**Fig. 7:**
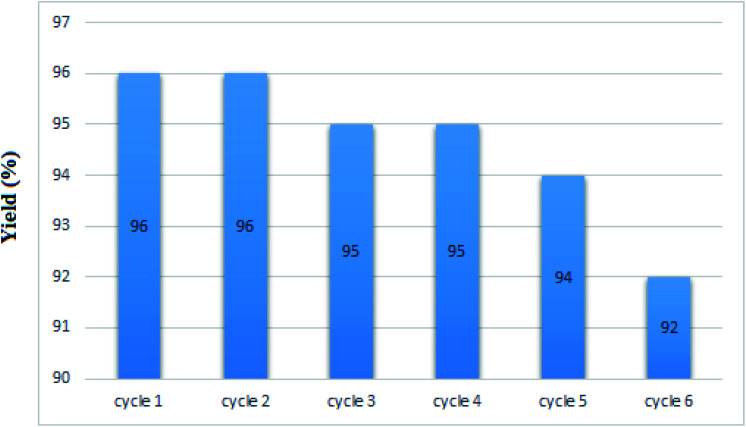
Recycling of nano-MgFe_2_O_4_/Cu in the synthesis of 2-phenyl-2-(4-phenyl-1*H*-1,2,3-triazol-1-yl)ethane-1-thiol.

**Fig. 8:**
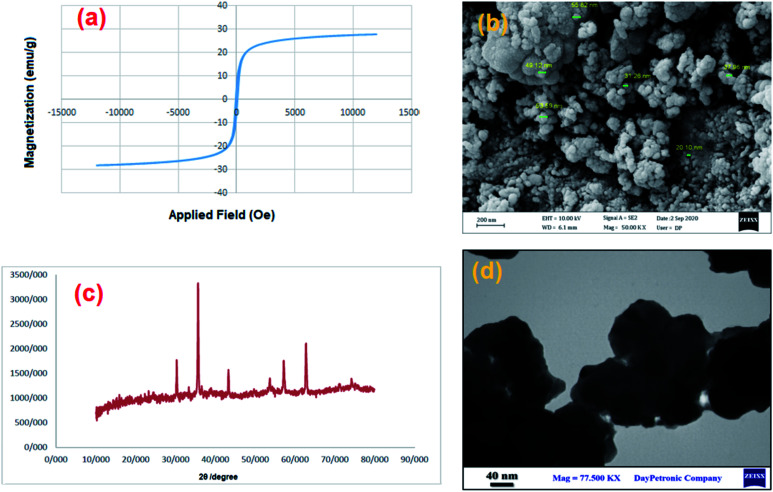
(a) Magnetization curve, (b) FESEM, (c) XRD pattern and (d) TEM image of MgFe_2_O_4_/Cu after five runs in the synthesis of 2-phenyl-2-(4-phenyl-1*H*-1,2,3-triazol-1-yl)ethane-1-thiol.

The recycling of the green nanocatalyst was investigated under the optimized reaction conditions (Table 2, entry 1). The nanoparticles were easily accumulated by applying an external magnetic field, washed with ethyl acetate and distilled water and, after drying, reused several times without any significant loss of activity (Fig. 7). The structure of the recovered catalyst was confirmed using VSM, FESEM, XRD and TEM analyses after five runs (Fig. 8).

The extent of Mg, Fe and Cu leaching during catalytic reaction was studied by ICP-OES analysis of the supernatant liquid after removal of catalyst and the result showed no presence of Mg, Fe and Cu in supernatant liquid.

### 3.4. Mercury poisoning and hot filtration tests

In order to confirm the heterogeneity of the catalyst, both hot filtration and mercury poisoning tests were performed. Accordingly, the filtration of the catalyst was carried out after 30 min at 100 °C and the filtrate was allowed to react for additional 2 hours, but the reaction due to the absence of copper did not take place, and no cyclization reaction was occurred.

The Hg poisoning test was conducted for the model reaction under the optimum conditions as follows: the one-pot reaction of styrene episulfide (1 mmol), phenyl acetylene (1 mmol), sodium azide (1.2 mmol) and MgFe_2_O_4_/Cu nanocatalyst (0.02 g) was carried out in the presence of Hg(0) excess (1.89 g, 9.43 mmol, 277 equiv.) under intense stirring conditions at 60 °C for 3 h in water. The suppression of catalysis by mercury is evidence for a heterogeneous catalyst.^58^ The added Hg(0) poisoned and inactivated MgFe_2_O_4_/Cu heterogeneous catalyst through amalgamating the metal catalyst or adsorbing on its surface and no product was obtained after 3 h.

### 3.5. The proposed mechanism for synthesis of β-thiol-1,4-disubstituted-1,2,3-triazoles catalyzed by MgFe_2_O_4_/Cu

The designed mechanism for the synthesis of β-thiol-1,4-disubstituted-1,2,3-triazole may comprise two possible pathways (A and B). MgFe_2_O_4_/Cu nanoparticles catalyze both cleavage of the thiirane ring and 1,3-dipolar cycloaddition leading to the formation of triazoles.^27,28^ First, as a result of non-covalent interaction a bond is formed between metal and azide, followed by activation of thiirane ring with MgFe_2_O_4_/Cu catalyst. Then, ring opening of thiirane is accomplished through azide transference from the catalyst which leads to the formation of 2-azido-2-arylethanethiol (pathway A). The thiirane rings carrying aryl groups due to the stability of benzyl carbocation prefer to be opened from the more hindered position *via* S_N_1 type of mechanism (α-cleavage); nevertheless, the regioselective ring opening of thiiranes bearing alkyl and allyl substituents by azide is powerfully preferred from less hindered carbon of the thiirane *via* S_N_2 type of mechanism (β-cleavage). In order to accredit the catalytic role of MgFe_2_O_4_/Cu in the pathway A, the styrene episulfide and sodium azide were reacted in the absence of catalyst, and it was found that, only a trace amount of 2-azido-2-arylethanethiol had been generated. For pathway A, consumption of styrene episulfide and sodium azide and also the generation of 2-azido-2-phenylethanethiol intermediate were monitored by gas chromatography (GC) analysis and thin layer chromatography (TLC) runs of the reaction mixture, and we found that 2-azido-2-arylethanethiol is formed easily (within the first 30 min of the reaction) and the rate determining step (RDS) was found to be the 1,3-dipolar cycloaddition step. 2-Azido-2-arylethanethiol was characterized by FT-IR spectrum and stretching frequency of 2097 cm^−1^ related to the azide (the FT-IR spectrum of 2-azido-2-phenylethanethiol has been provided in ESI Section†).

**Scheme 4:**
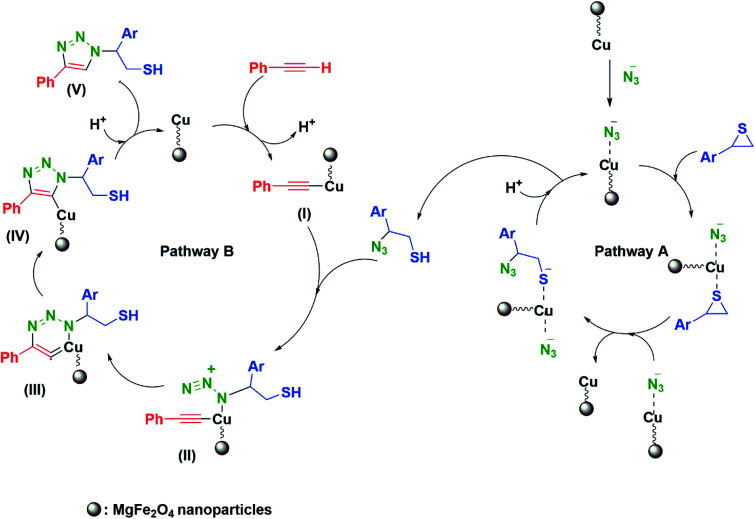
The mechanism proposed for the one-pot synthesis of β-thiol-1,4-disubstituted-1,2,3-triazole catalyzed by MgFe_2_O_4_/Cu.

The pathway B shows the insertion of copper to the C–H bond of phenylacetylene and generation of the intermediate(i), which accelerates the [3+2] cycloaddition between azide and carbon–carbon triple bond of *in situ* produced intermediate(ii), to afford the Cu–C-triazole(iv). The phenylacetylene consumption and also the disappearance of the 2-azido-2-arylethanethiol intermediate, were monitored by GC analysis and TLC runs of the reaction mixture. Eventually, proteolysis of the Cu–C bond of intermediate(iv) by aqueous media gives the corresponding β-thiol-1,4-disubstituted-1,2,3-triazole(v) (Scheme 4).

In order to evaluate the accuracy of reaction RDS, 2-azido-2-phenylethanethiol was separately reacted with phenylacetylene in the presence of MgFe_2_O_4_/Cu nanocatalyst. The formation of corresponding 1,2,3-triazole was monitored *via* GC analysis and TLC runs. It was observed that the reaction was carried out within 2 h. This result demonstrated that the pathway B determines the reaction rate.

To confirm the formation of acetylide intermediate(i), phenylacetylene and MgFe_2_O_4_/Cu nanocatalyst were mixed in a separate experiment in aqueous media, and pH of water as a solvent was investigated. A 0.6 unit decrease in pH after 20 min was detected, indicative of terminal proton release to the water, due to the initial coordination of phenylacetylene to copper to form acetylide intermediate(i).

## 4. Conclusions

In summary, in this research, the magnetic nanocomposite of MgFe_2_O_4_/Cu has been easily manufactured through a solid-state procedure and it was then characterized by different techniques such as VSM, FESEM, TEM, XRD, EDS and FT-IR. This novel composite has been utilized as an efficient catalyst for one-pot synthesis of β-thiol-1,4-disubstituted-1,2,3-triazoles as new products *via* three component reactions of sodium azide, terminal alkynes, and various thiiranes in water. The method reported is completely new due to the novelty of both the catalyst and the triazole products. Furthermore, perfect regioselectivity, the simple process, high product yields, short reaction times, the use of eco-friendly solvent, easy separation and recycling of catalyst are significant advantages of this proposed procedure.

## Conflicts of interest

There are no conflicts to declare.

## Supplementary Material

RA-011-D1RA01588E-s001
