# Supplementary material for: Preparation, characterization and application of MgFe2O4/Cu nanocomposite as a new magnetic catalyst for one-pot regioselective synthesis of β-thiol-1,4-disubstituted-1,2,3-triazoles
Source: RSC Adv. 2021 Apr 7;11(22):13061–76. doi: 10.1039/d1ra01588e (PMC8697271; doi:10.1039/d1ra01588e)
Supplement: RA-011-D1RA01588E-s001 [file RA-011-D1RA01588E-s001.pdf]

Electronic Supplementary Material (ESI) for RSC Advances.
This journal is © The Royal Society of Chemistry 2021

Supplementary Information

Preparation, characterization and application of $MgFe_2O_4$/Cu nanocomposite as a new magnetic catalyst for one-pot regioselective synthesis of *β*-thiol-1,4-disubstituted-1,2,3-triazoles

Ronak Eisavi* and Kazhal Naseri

Department of Chemistry, Payame Noor Universtiy, PO BOX 19395-3697, Tehran, Iran

Email: roonak.isavi@gmail.com

FT-IR, ^{1}H NMR and ^{13}C NMR spectra of β-Thiol-1,4-disubstituted-1,2,3-triazoles ***(1-11)b*** *and FT-IR spectrum of 2-azido-2-phenylethanthiol intermediate are presented as following:*

2-Phenyl-2-(4-phenyl-1H-1,2,3-triazol-1-yl)ethane-1-thiol (1b)

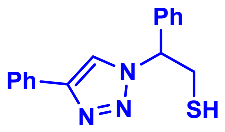

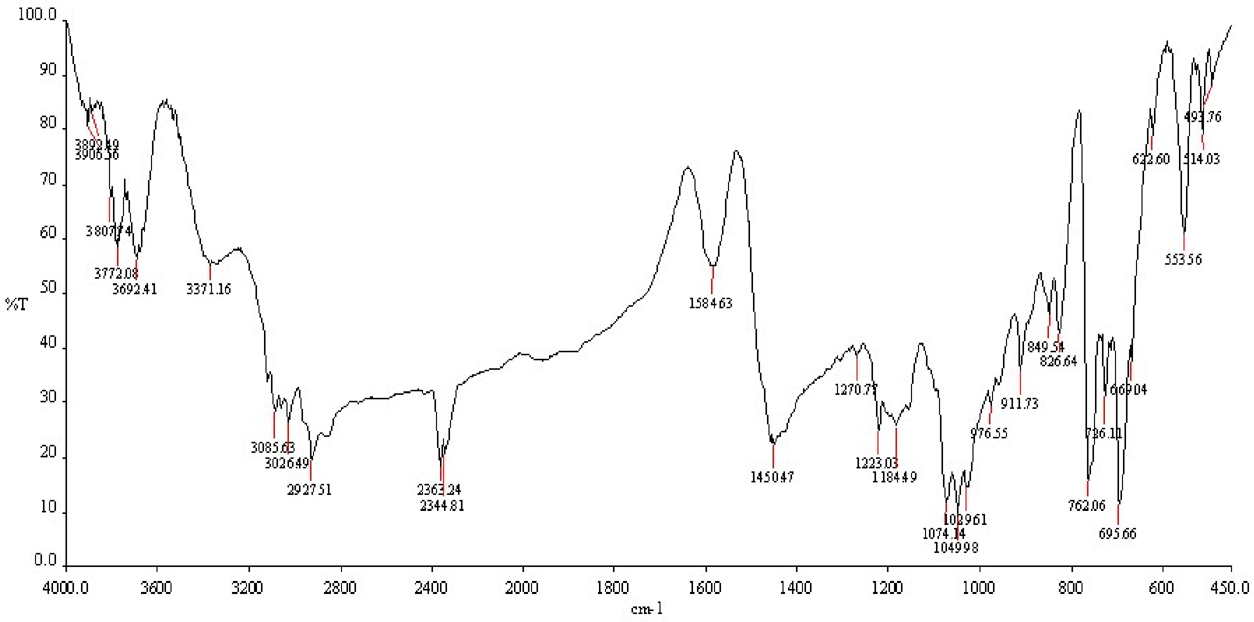

Figure S1. FT-IR Spectrum of 2-phenyl-2-(4-phenyl-1*H*-1,2,3-triazol-1-yl)ethane-1-thiol (1b)

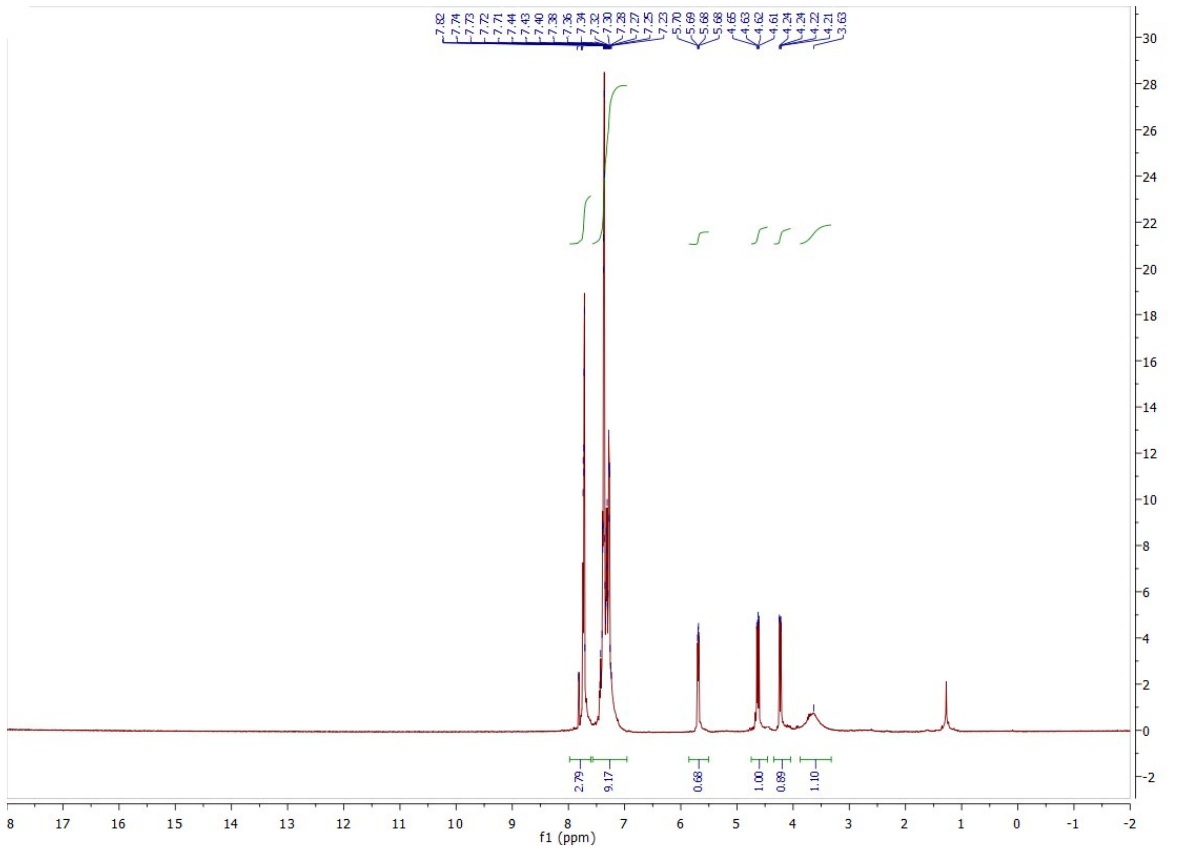

Figure S2. **^{1}H NMR Spectrum of 2-phenyl-2-(4-phenyl-1*H*-1,2,3-triazol-1-yl)ethane-1-thiol (1b)**

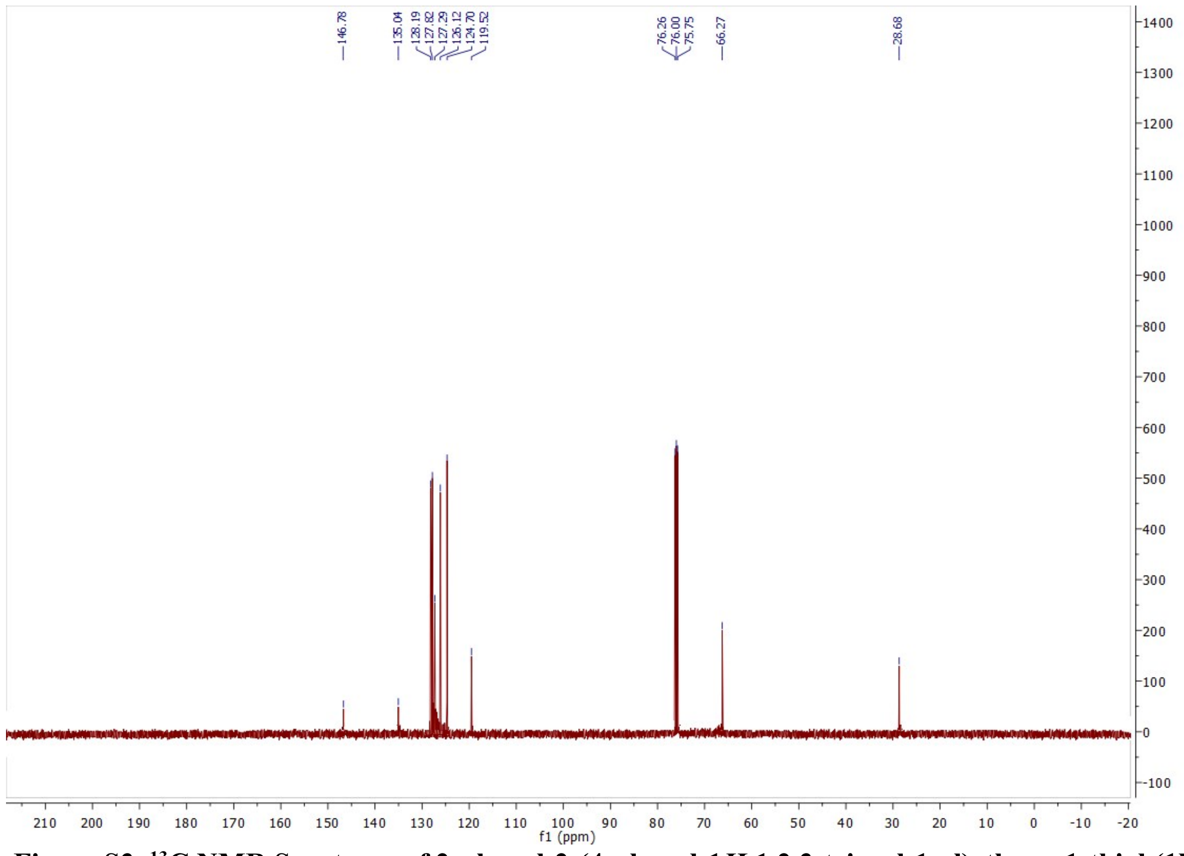

Figure S3. **^{13}C NMR Spectrum of 2-phenyl-2-(4-phenyl-1*H*-1,2,3-triazol-1-yl)ethane-1-thiol (1b)**

1-Phenoxy-3-(4-phenyl-1H-1,2,3-triazol-1-yl)propane-2-thiol (2b)

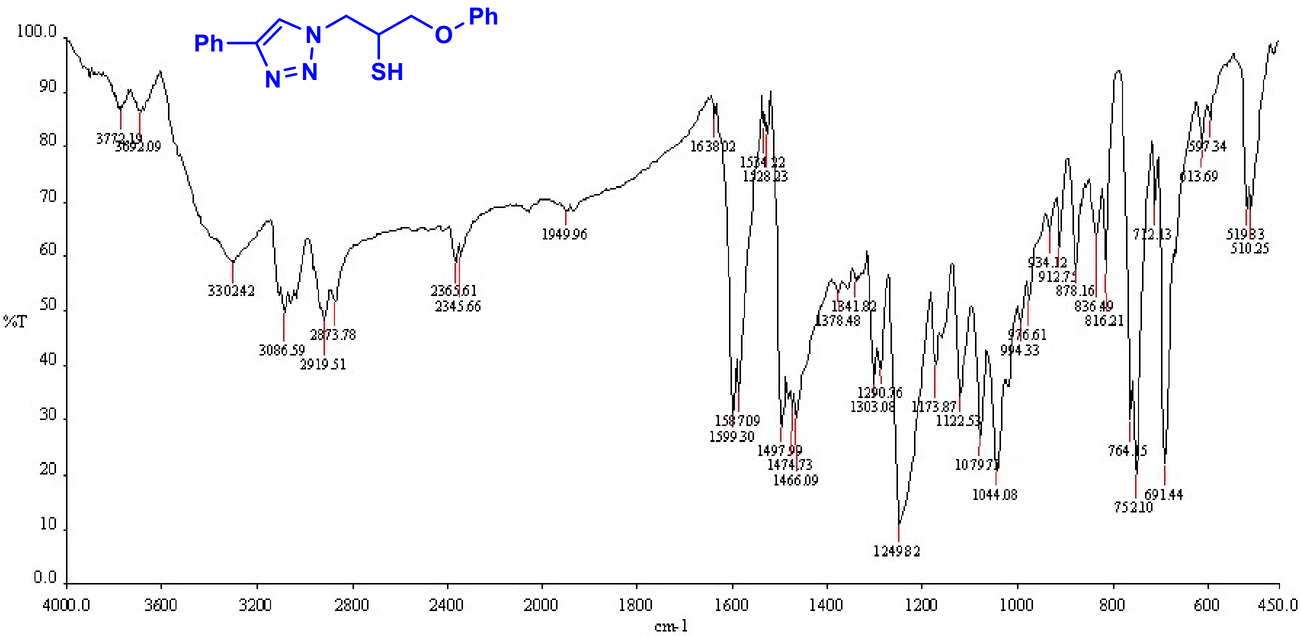

Figure S4. FT-IR Spectrum of 1-phenoxy-3-(4-phenyl-1*H*-1,2,3-triazol-1-yl)propane-2-thiol (2b)

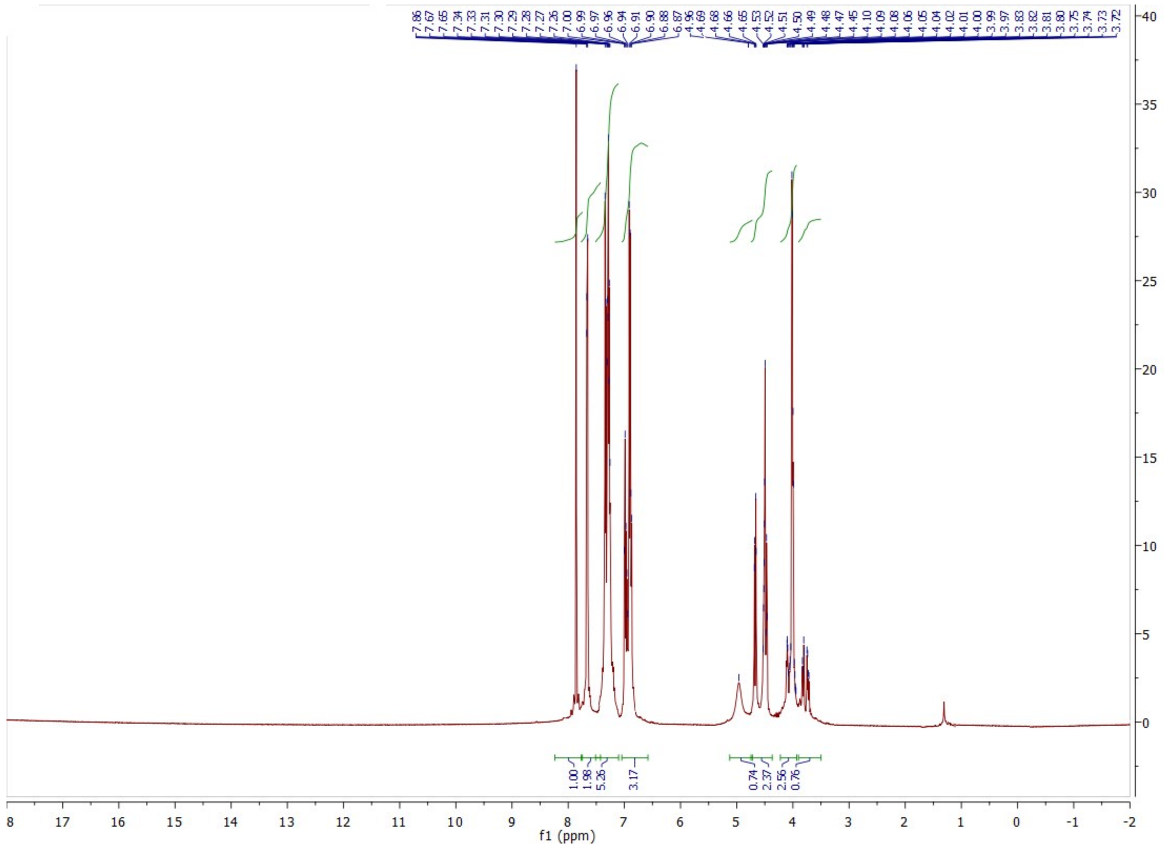

Figure S5. ^{1}H NMR Spectrum of 1-phenoxy-3-(4-phenyl-1*H*-1,2,3-triazol-1-yl)propane-2-thiol (2b)

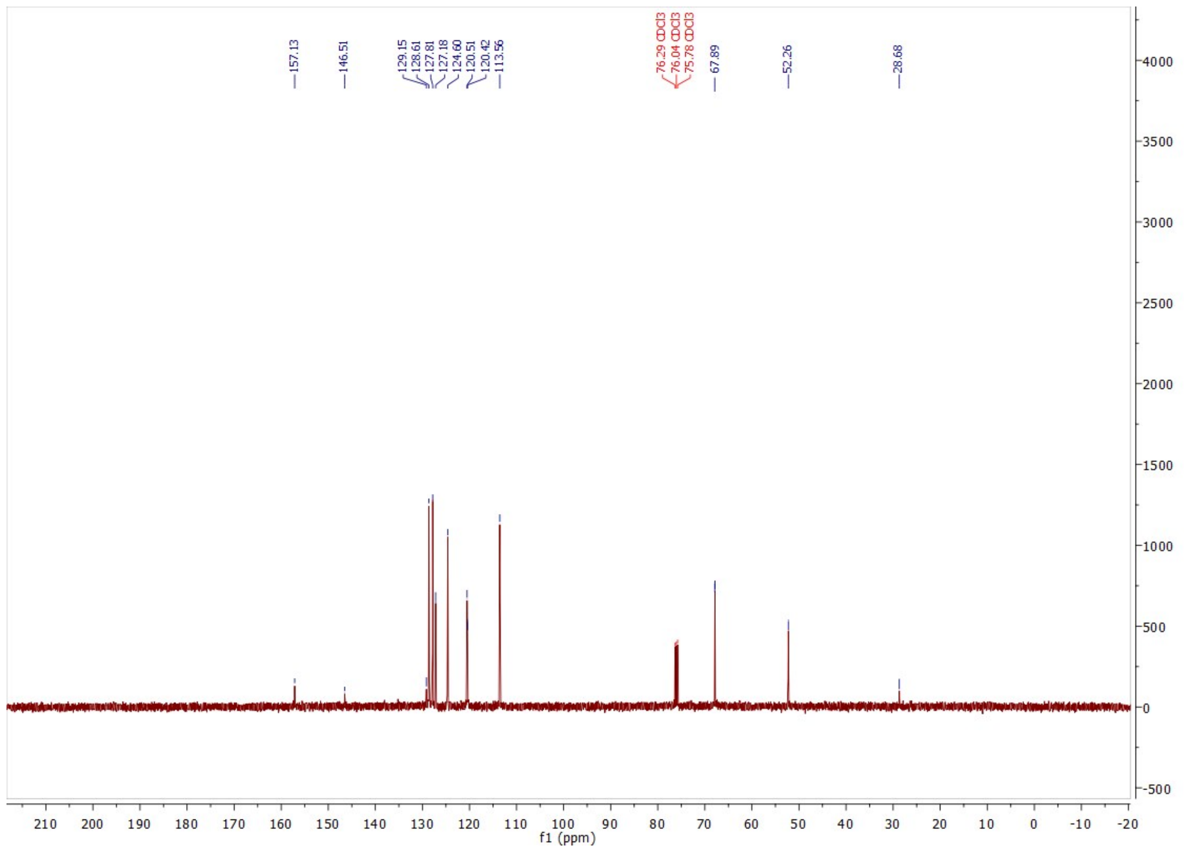

Figure S6. ^{13}C NMR Spectrum of 1-phenoxy-3-(4-phenyl-1*H*-1,2,3-triazol-1-yl)propane-2-thiol (2b)

1-Chloro-3-(5-phenyl-1H-1,2,3-triazol-1-yl)propane-2-thiol (3b)

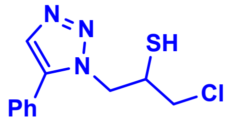

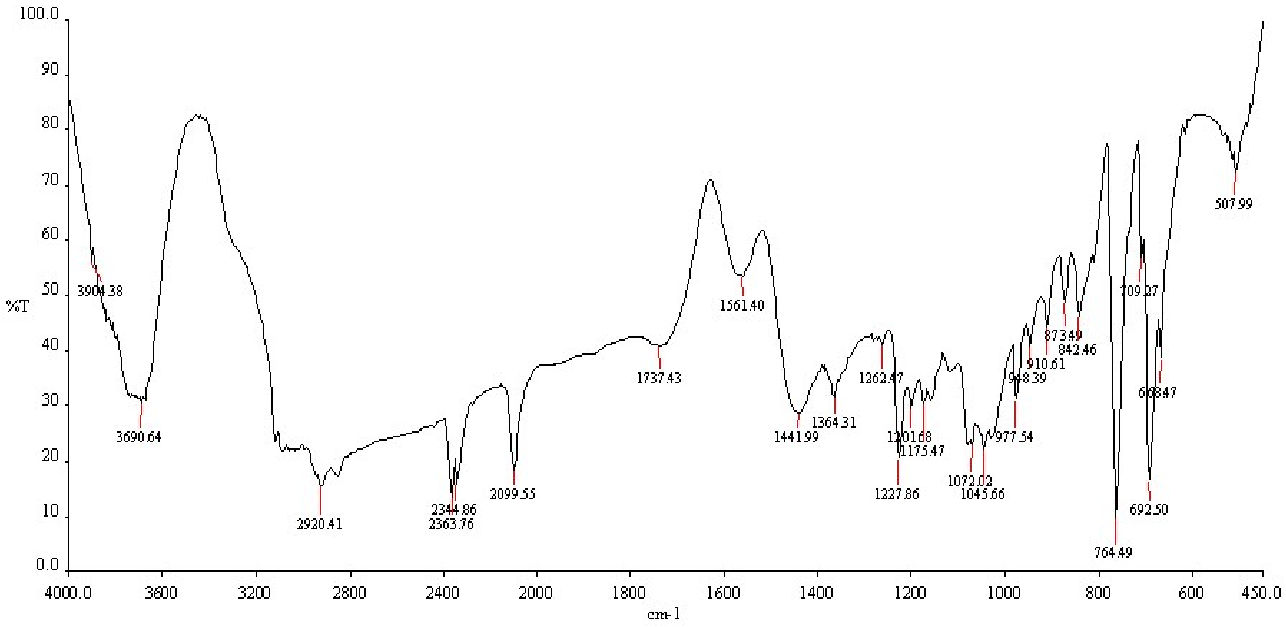

Figure S7. FT-IR Spectrum of 1-chloro-3-(5-phenyl-1H-1,2,3-triazol-1-yl)propane-2-thiol (3b)

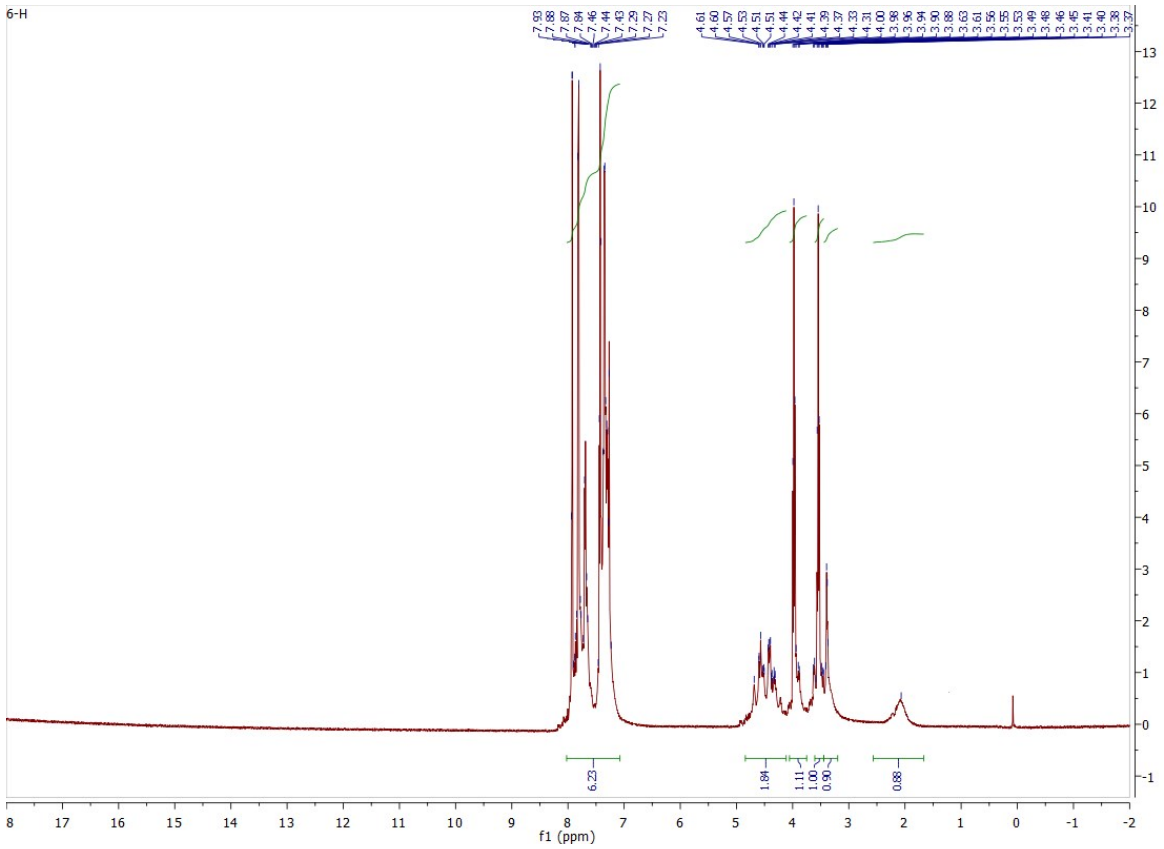

Figure S8. ^{1}H NMR Spectrum of 1-chloro-3-(5-phenyl-1H-1,2,3-triazol-1-yl)propane-2-thiol (3b)

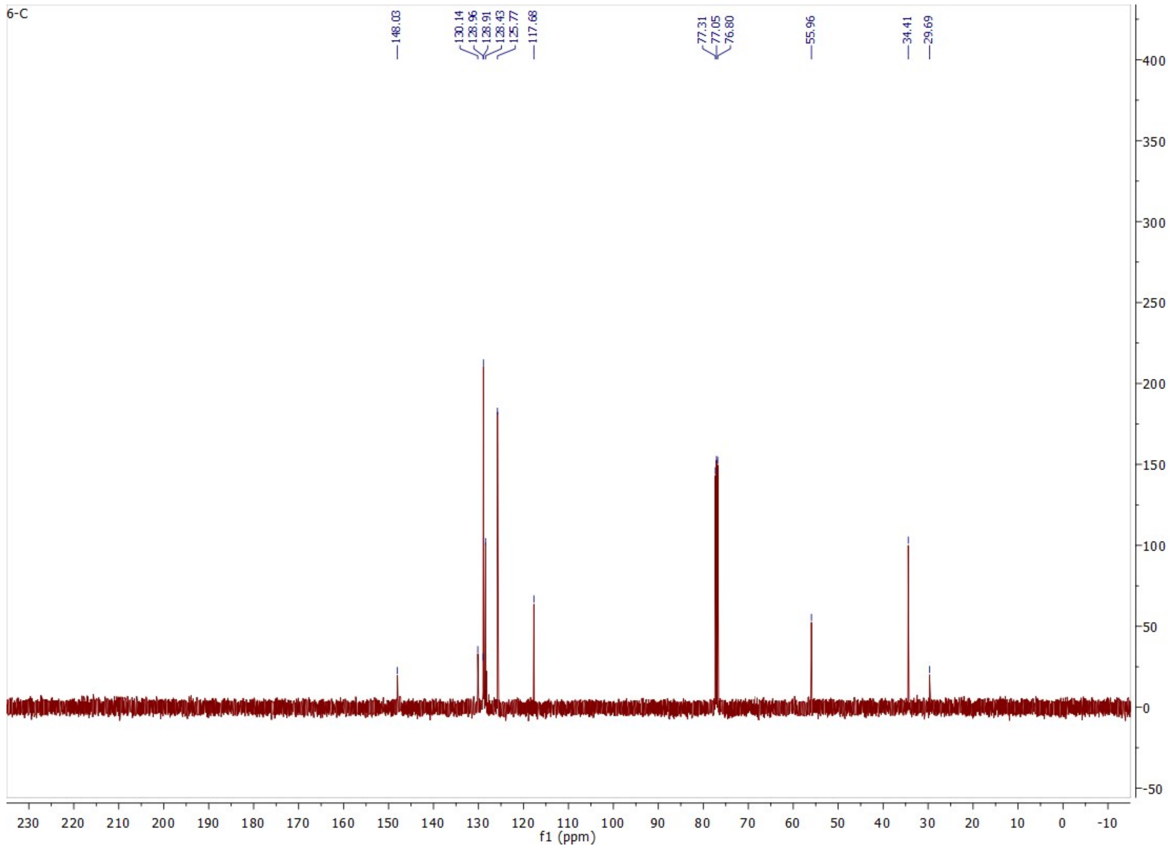

Figure S9. ^{13}C NMR Spectrum of 1-chloro-3-(5-phenyl-1H-1,2,3-triazol-1-yl)propane-2-thiol (3b)

1-(4-Phenyl-1H-1,2,3-triazol-1-yl)butane-2-thiol (4b)

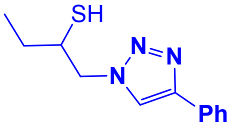

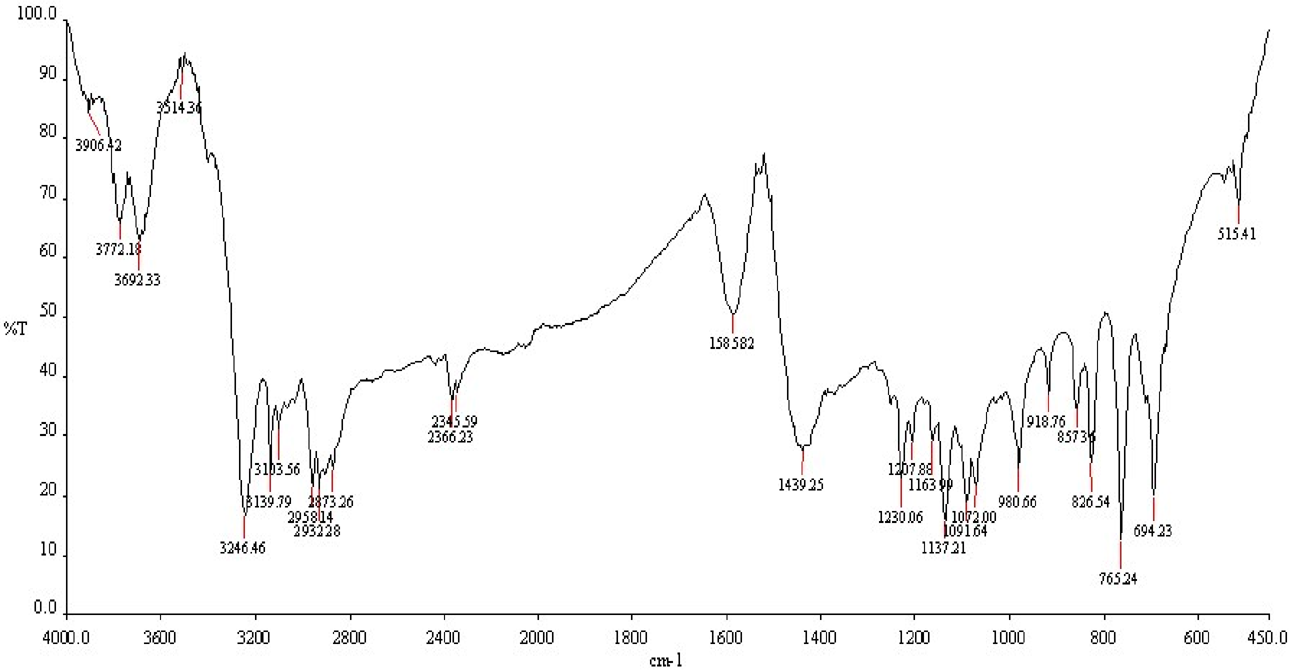

Figure S10. FT-IR Spectrum of 1-(4-phenyl-1*H*-1,2,3-triazol-1-yl)butane-2-thiol (4b)

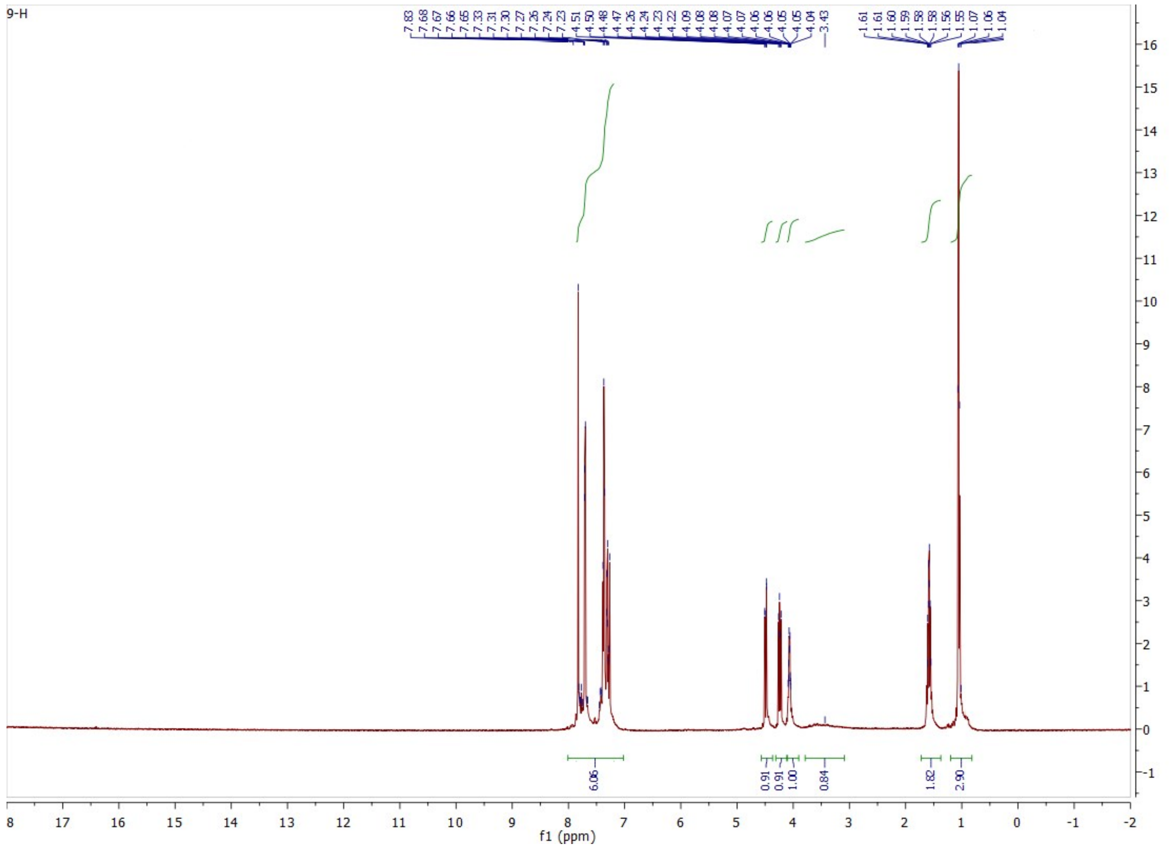

Figure S11. ^{1}H NMR Spectrum of 1-(4-phenyl-1*H*-1,2,3-triazol-1-yl)butane-2-thiol (4b)

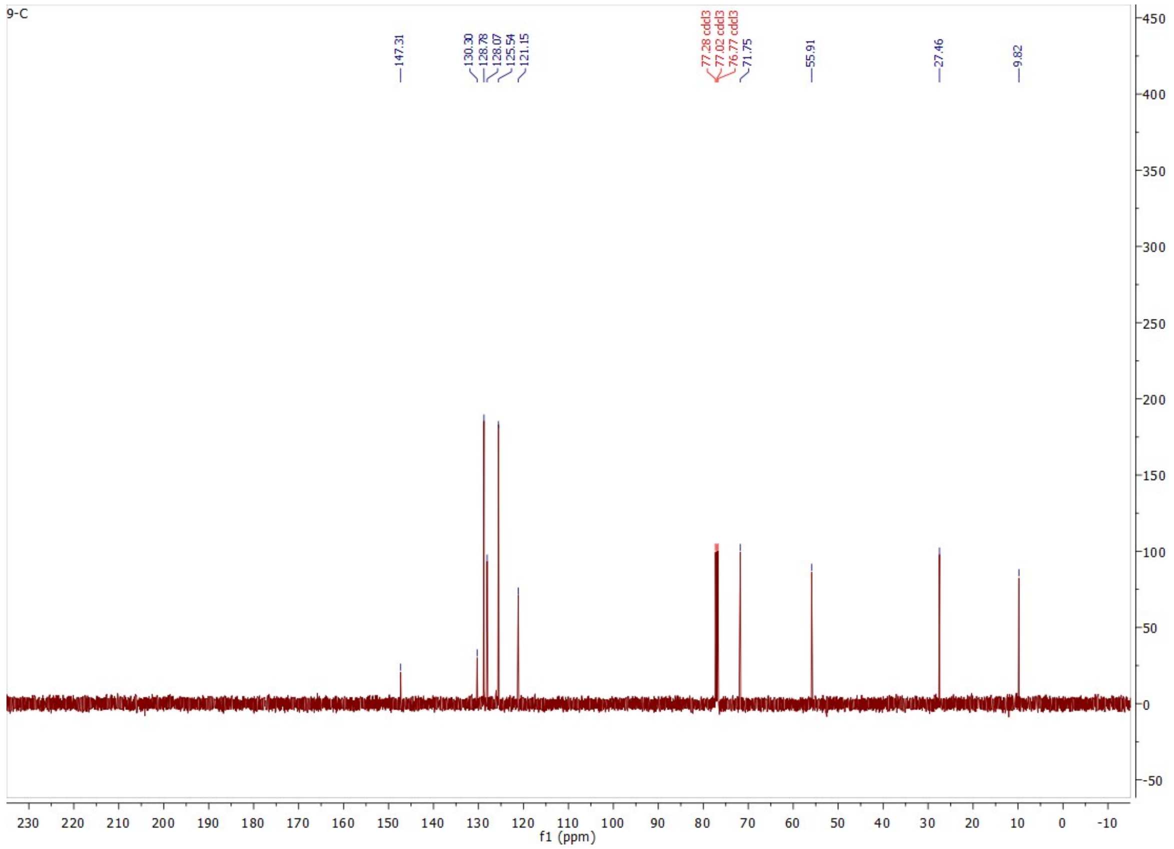

Figure S12. ^{13}**C NMR Spectrum of 1-(4-phenyl-1*H*-1,2,3-triazol-1-yl)butane-2-thiol (4b)**

1-Isopropoxy-3-(4-phenyl-1H-1,2,3-triazol-1-yl)propane-2-thiol (5b)

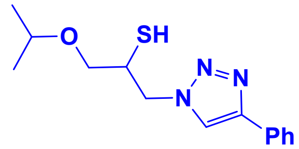

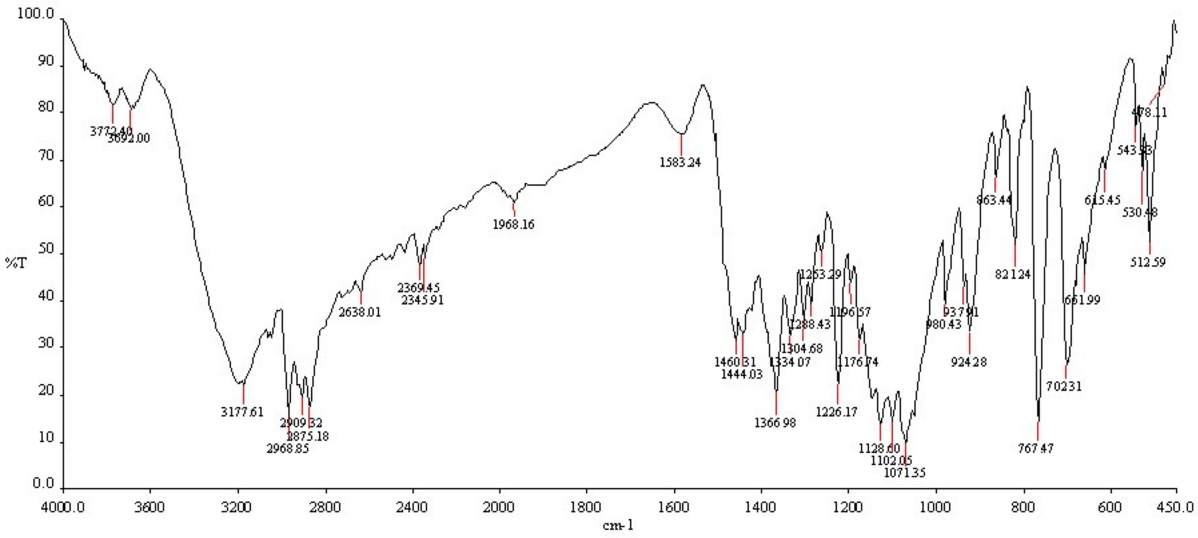

Figure S13. FT-IR Spectrum of 1-isopropoxy-3-(4-phenyl-1*H*-1,2,3-triazol-1-yl)propane-2-thiol (5b)

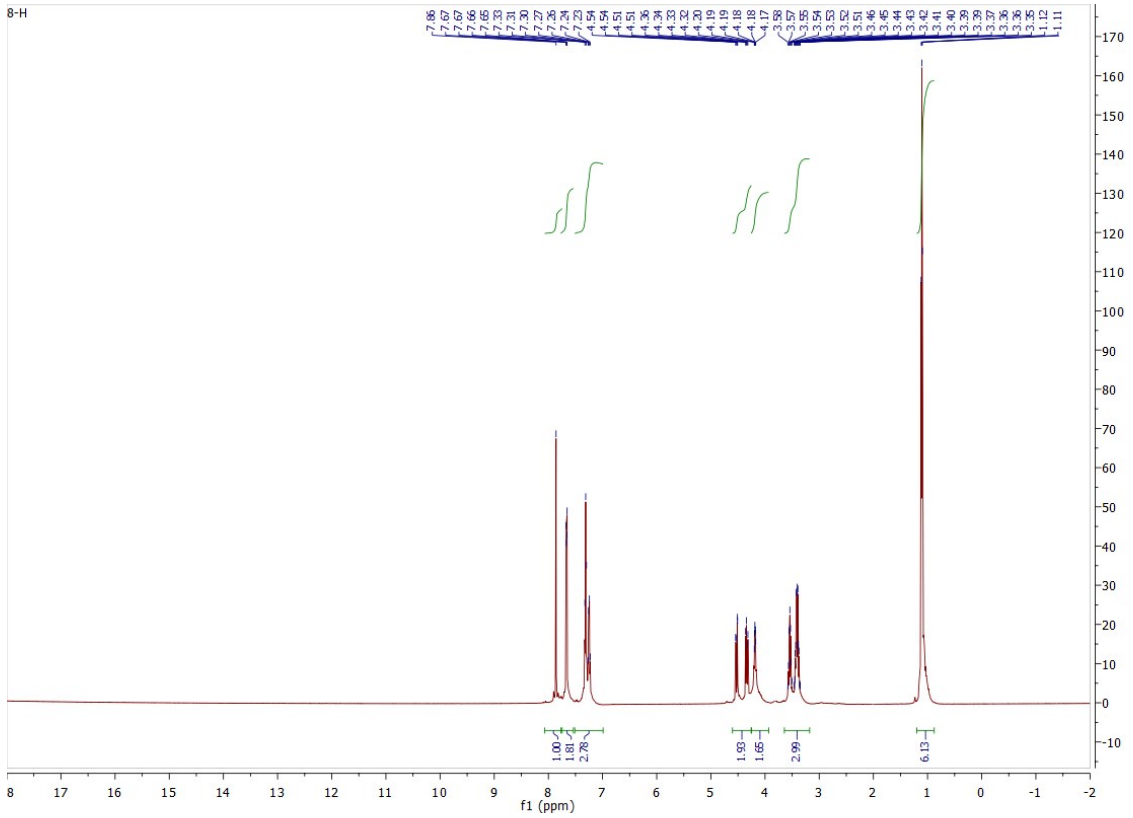

Figure S14. ¹H NMR Spectrum of 1-isopropoxy-3-(4-phenyl-1*H*-1,2,3-triazol-1-yl)propane-2-thiol (5b)

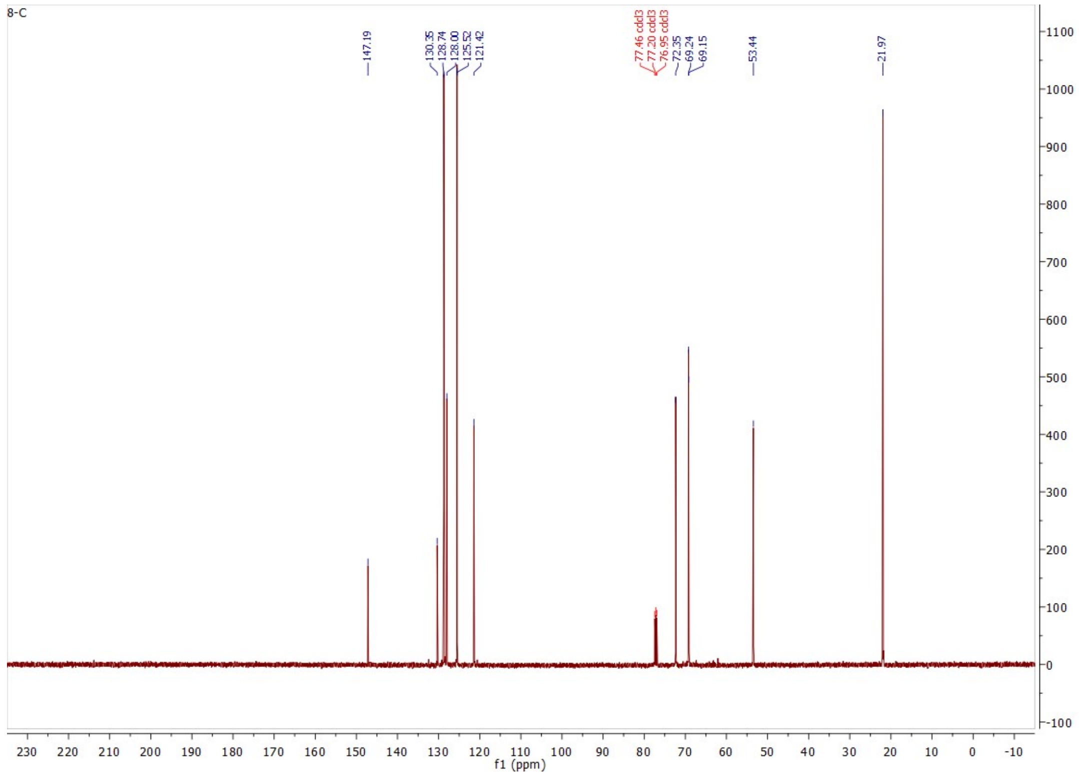

Figure S15. ¹³C NMR Spectrum of 1-isopropoxy-3-(4-phenyl-1*H*-1,2,3-triazol-1-yl)propane-2-thiol (5b)

2-(4-Phenyl-1H-1,2,3-triazol-1-yl)cyclohexane-1-thiol (6b)

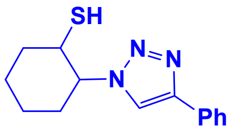

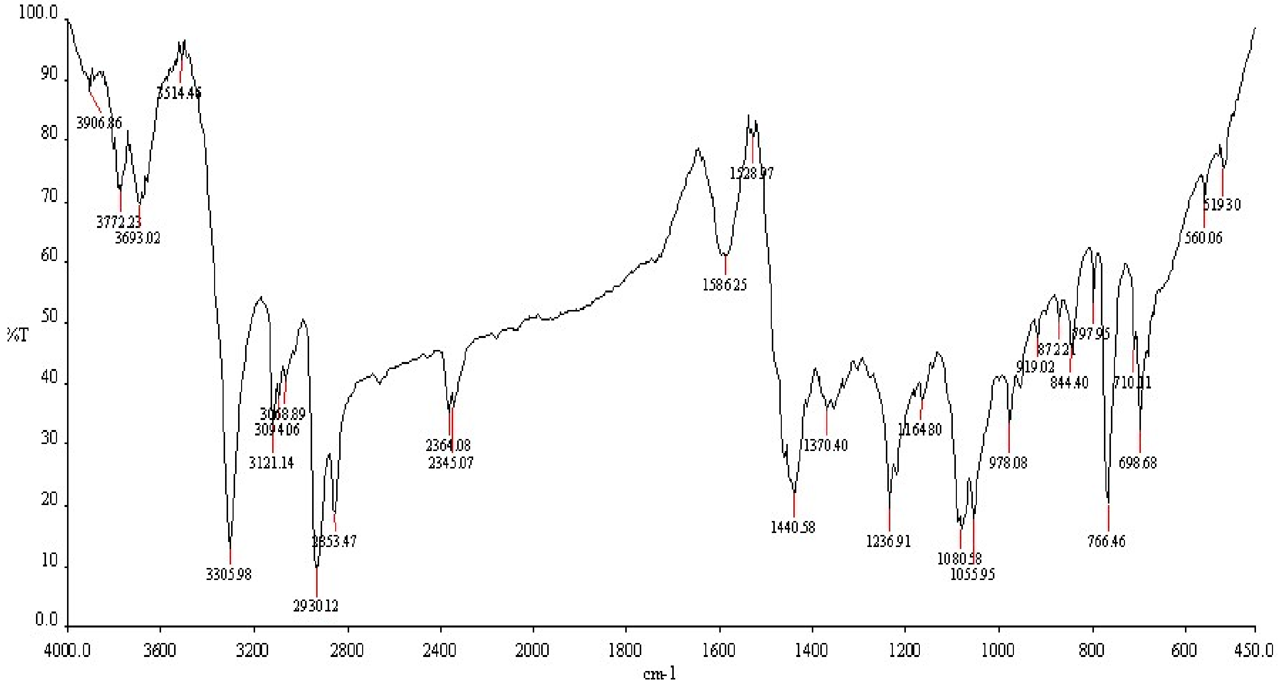

Figure S16. FT-IR Spectrum of 2-(4-phenyl-1*H*-1,2,3-triazol-1-yl)cyclohexane-1-thiol (6b)

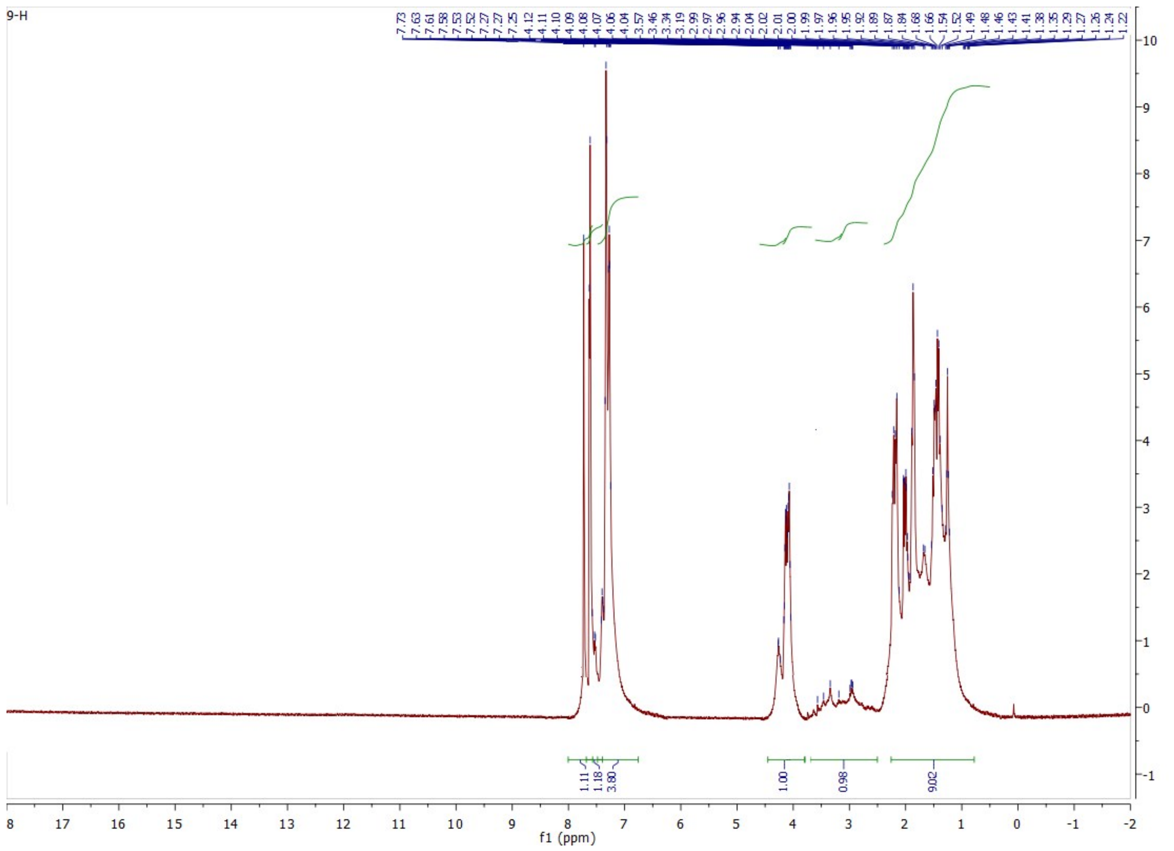

Figure S17. ^{1}H NMR Spectrum of 2-(4-phenyl-1*H*-1,2,3-triazol-1-yl)cyclohexane-1-thiol (6b)

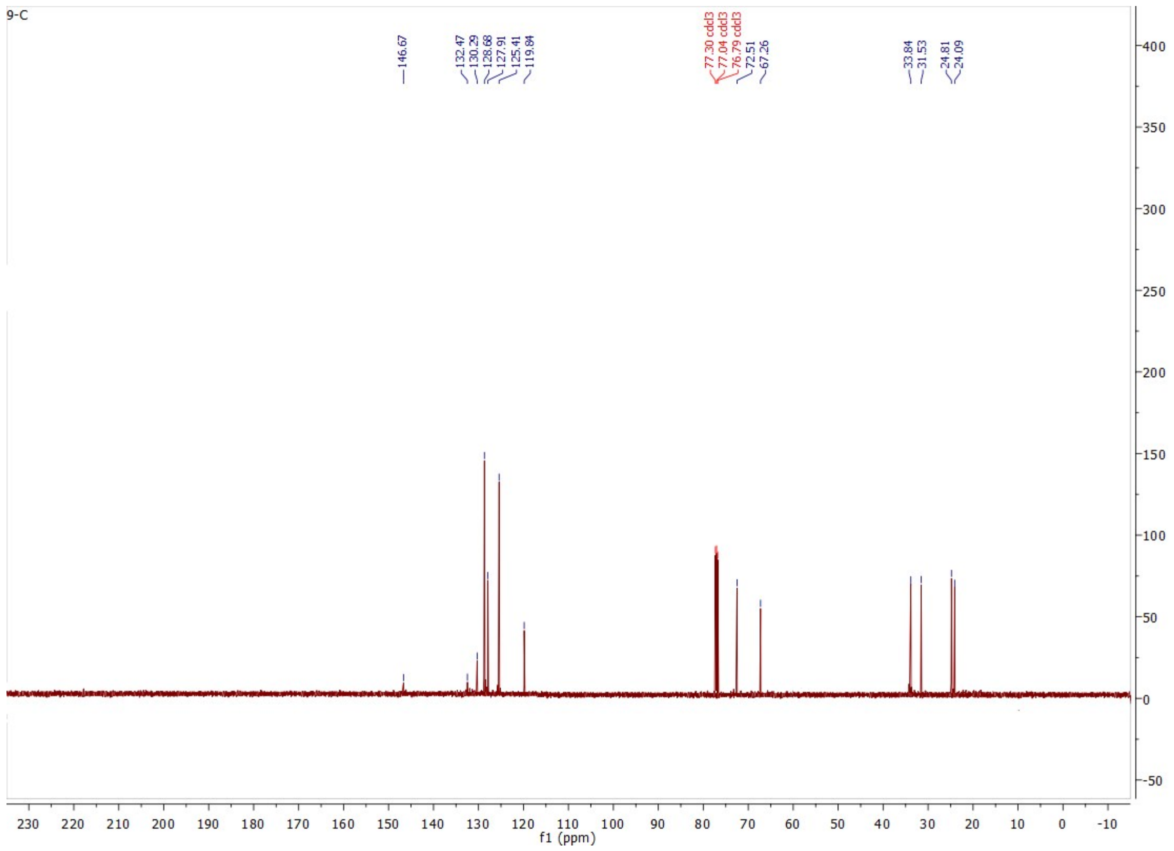

Figure S18. ^{13}C NMR Spectrum of 2-(4-phenyl-1*H*-1,2,3-triazol-1-yl)cyclohexane-1-thiol (6b)

1-(Allyloxy)-3-(4-phenyl-1H-1,2,3-triazol-1-yl)propane-2-thiol (7b)

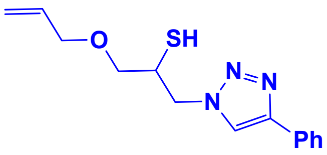

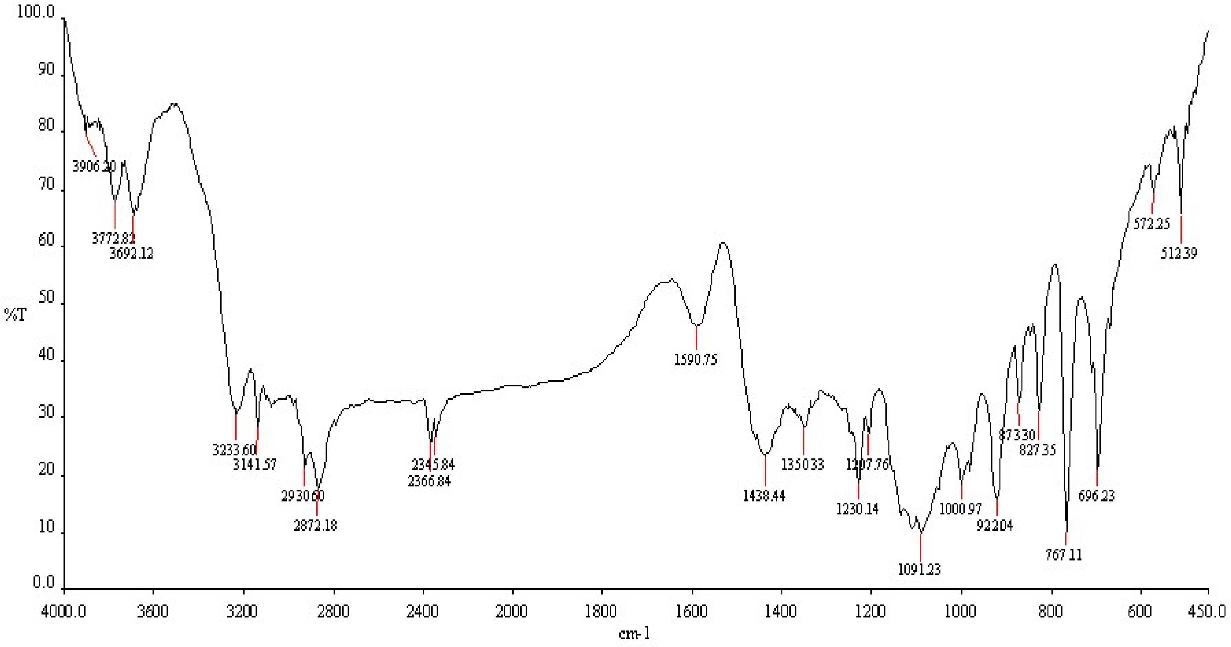

Figure S19. FT-IR Spectrum of 1-(allyloxy)-3-(4-phenyl-1*H*-1,2,3-triazol-1-yl)propane-2-thiol (7b)

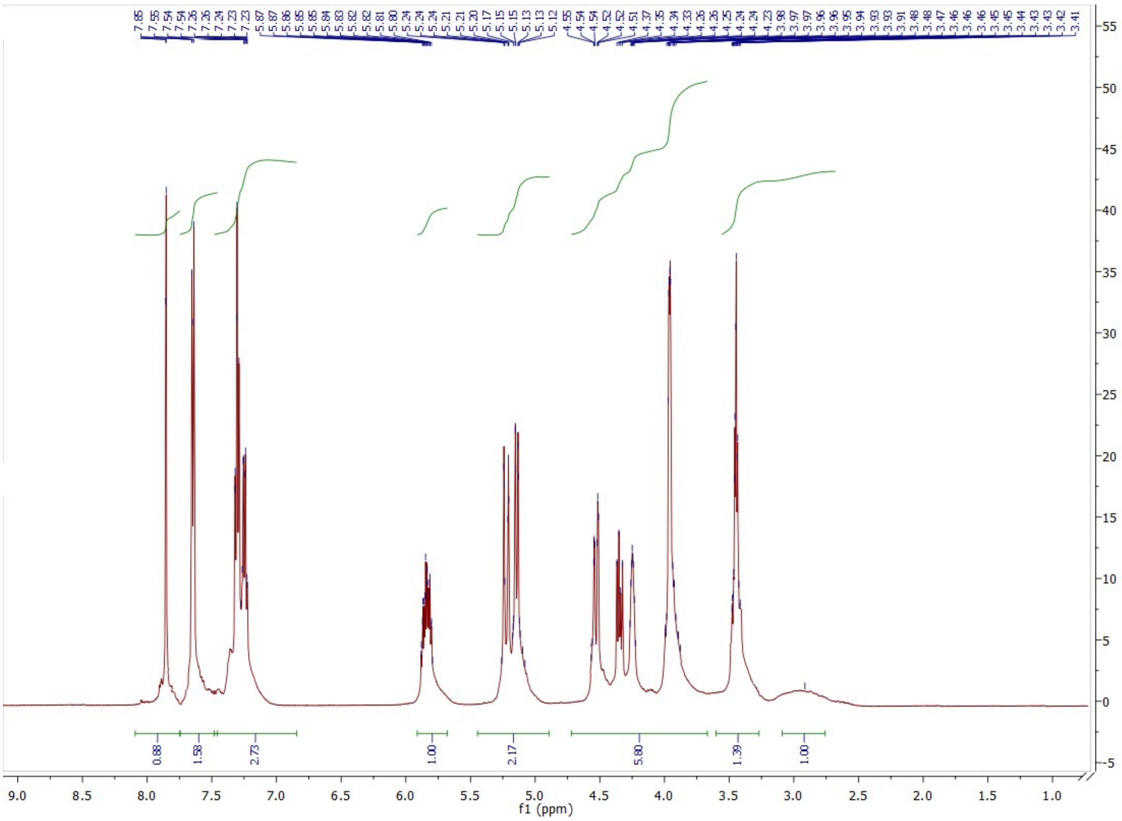

Figure S20. ^{1}**H NMR Spectrum of 1-(allyloxy)-3-(4-phenyl-1*H*-1,2,3-triazol-1-yl)propane-2-thiol (7b)**

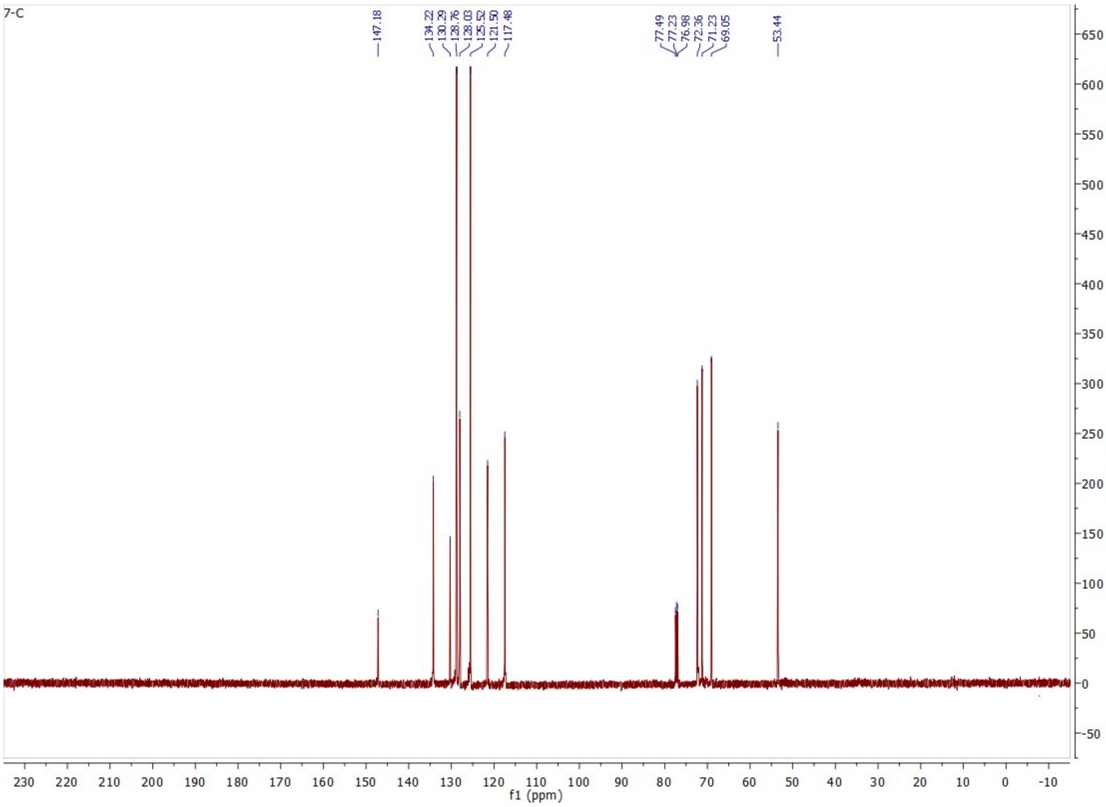

Figure S21. ^{13}**C NMR Spectrum of 1-(allyloxy)-3-(4-phenyl-1*H*-1,2,3-triazol-1-yl)propane-2-thiol (7b)**

2-Mercapto-3-(4-phenyl-1H-1,2,3-triazol-1-yl)propyl methacrylate (8b)

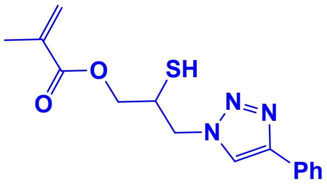

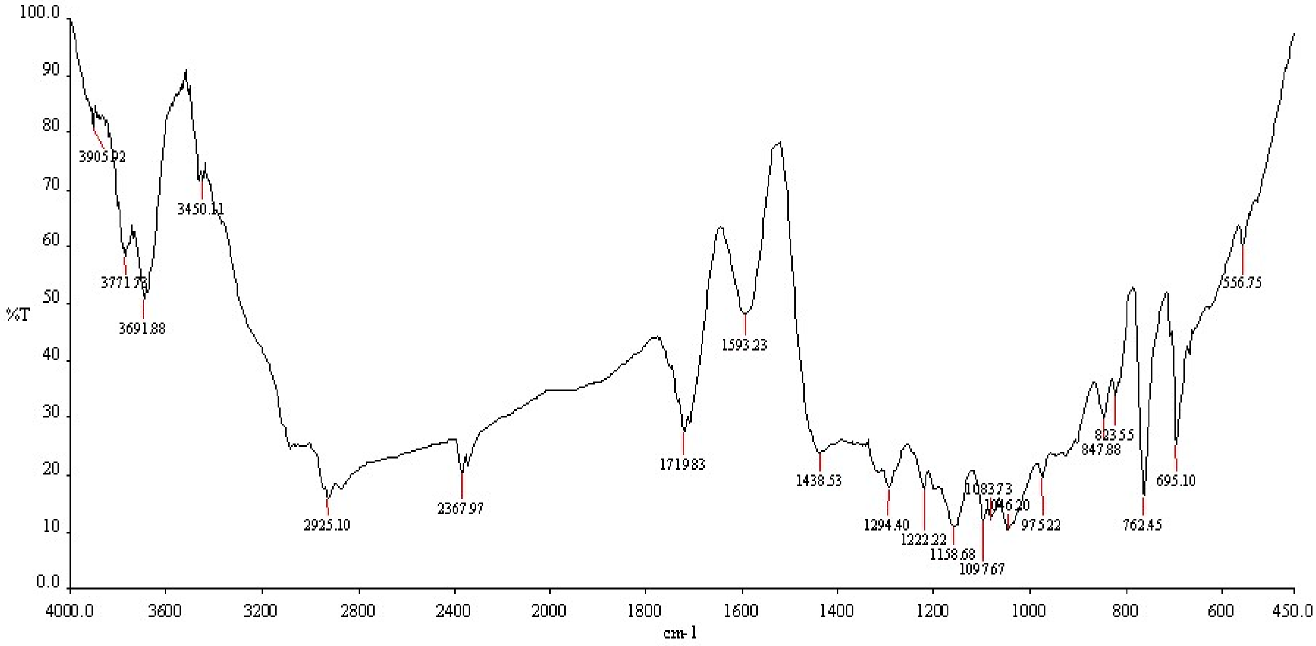

Figure S22. FT-IR Spectrum of 2-mercapto-3-(4-phenyl-1*H*-1,2,3-triazol-1-yl)propyl methacrylate (8b)

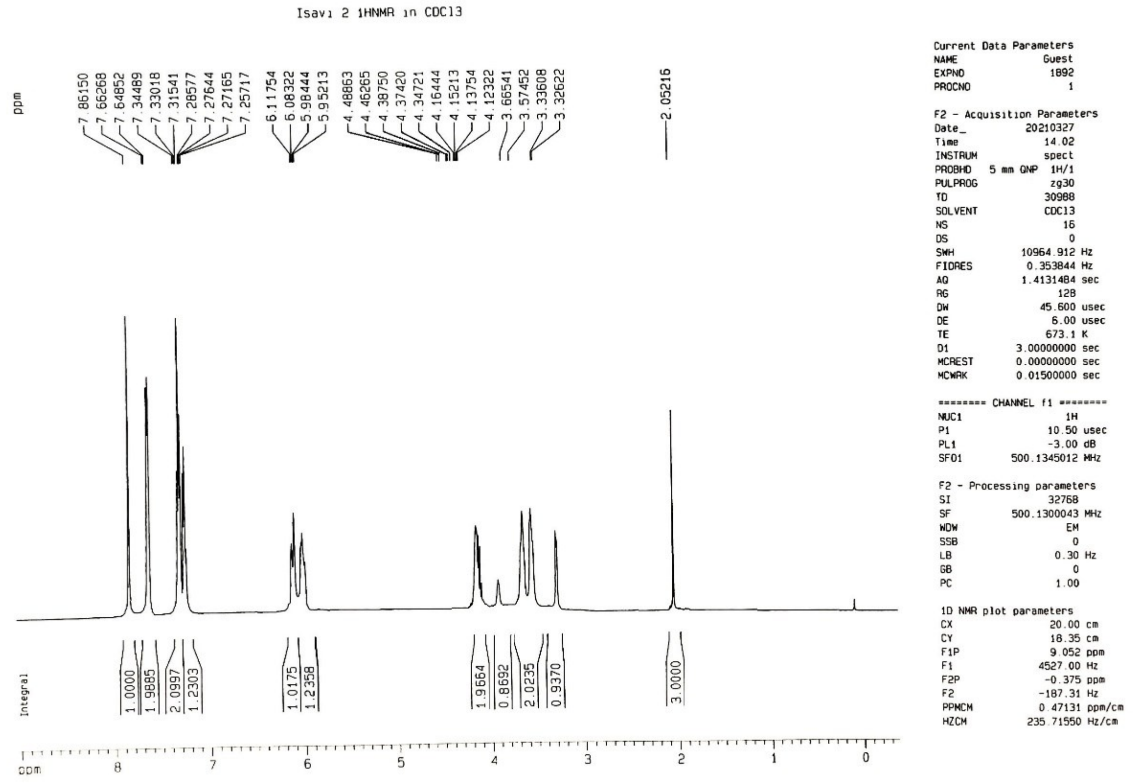

Figure S23. ¹H NMR Spectrum of 2-mercapto-3-(4-phenyl-1*H*-1,2,3-triazol-1-yl)propyl methacrylate (8b)

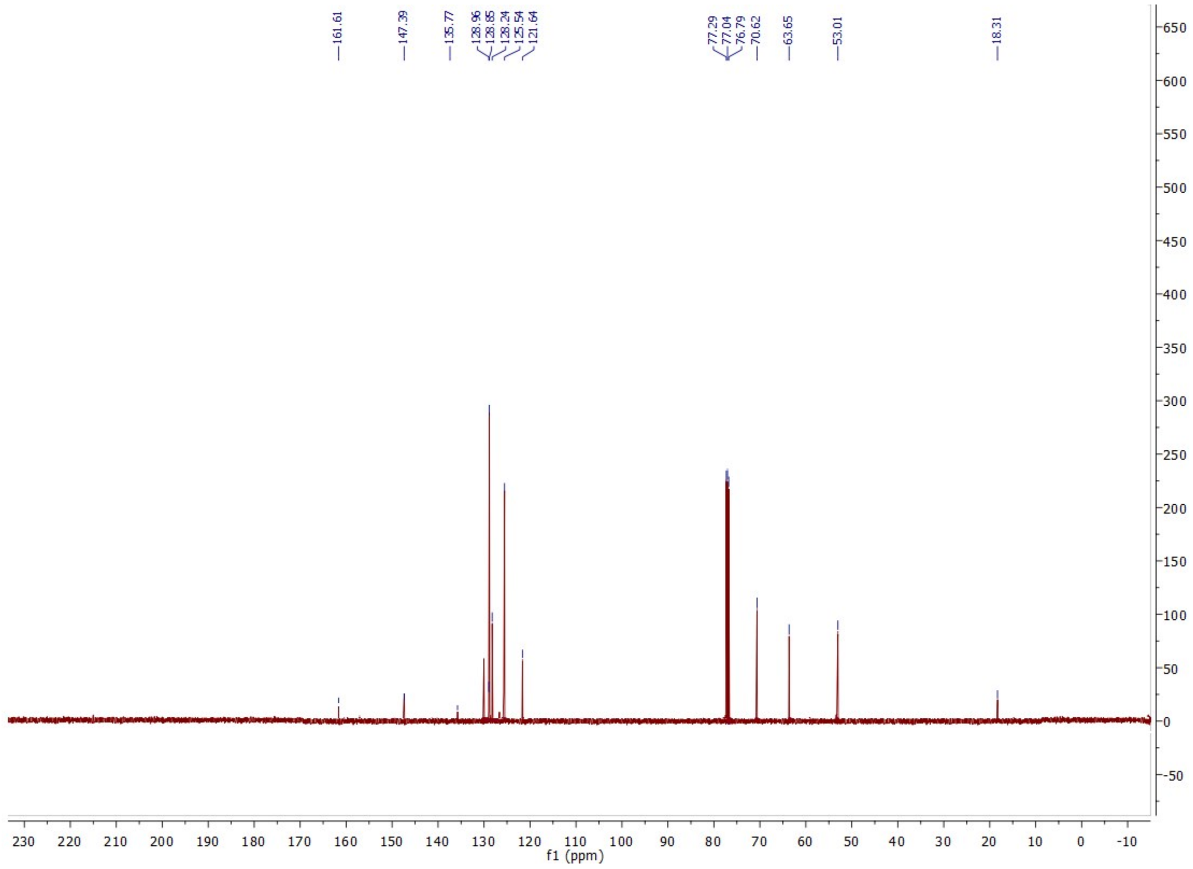

Figure S24. ^{13}C NMR Spectrum of 2-mercapto-3-(4-phenyl-1*H*-1,2,3-triazol-1-yl)propyl methacrylate (8b)

2-(4-Cyclohexyl-1H-1,2,3-triazol-1-yl)-2-phenylethane-1-thiol (9b)

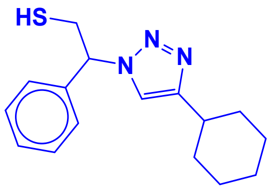

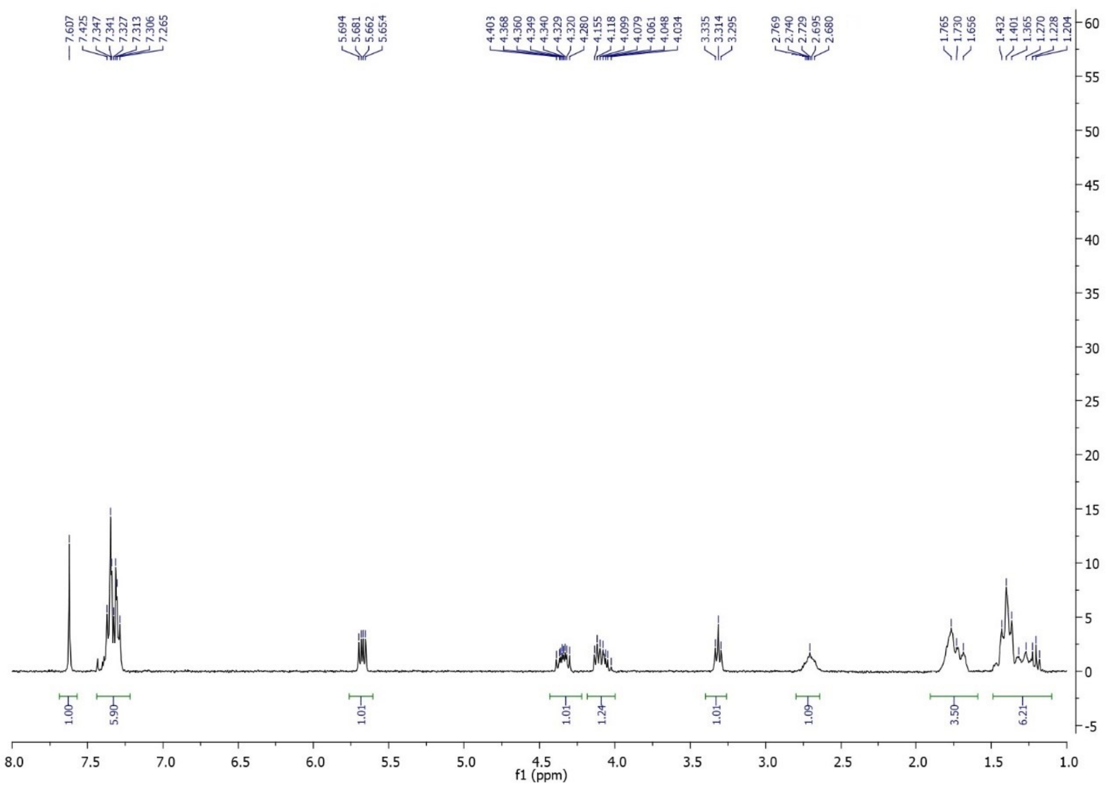

Figure S25. ^{1}H NMR Spectrum of 2-(4-cyclohexyl-1*H*-1,2,3-triazol-1-yl)-2-phenylethane-1-thiol (9b)

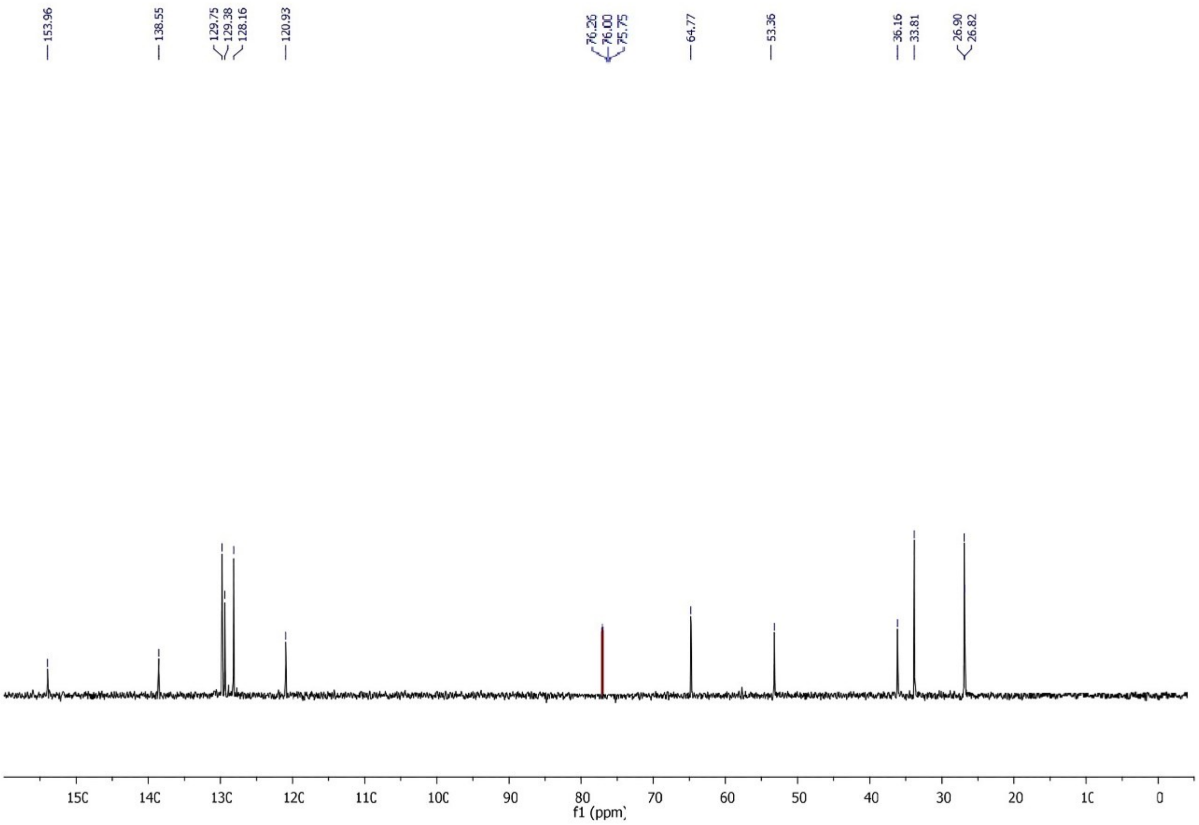

Figure S26. ^{13}**C NMR Spectrum of 2-(4-cyclohexyl-1*H*-1,2,3-triazol-1-yl)-2-phenylethane-1-thiol (9b)**

(1-(2-Mercapto-1-phenylethyl)-1H-1,2,3-triazol-4-yl)methanol (10b)

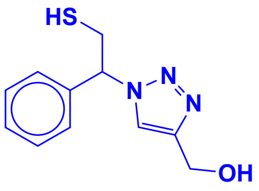

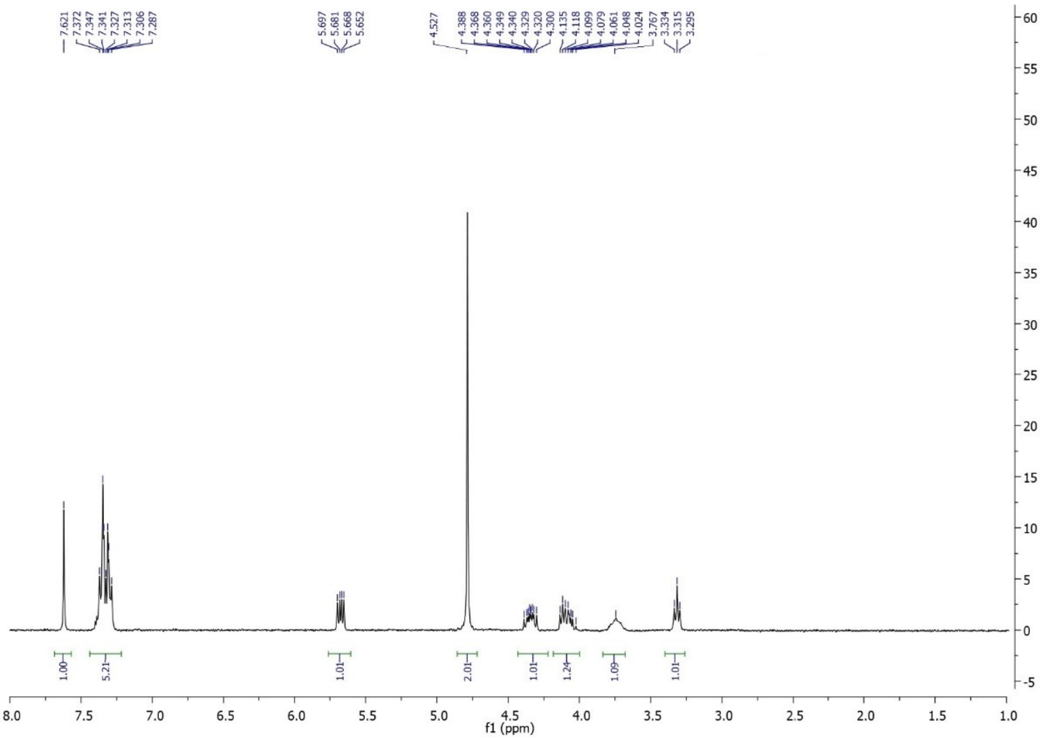

Figure S27. ^{1}H NMR Spectrum of (1-(2-mercapto-1-phenylethyl)-1*H*-1,2,3-triazol-4-yl)methanol (10b)

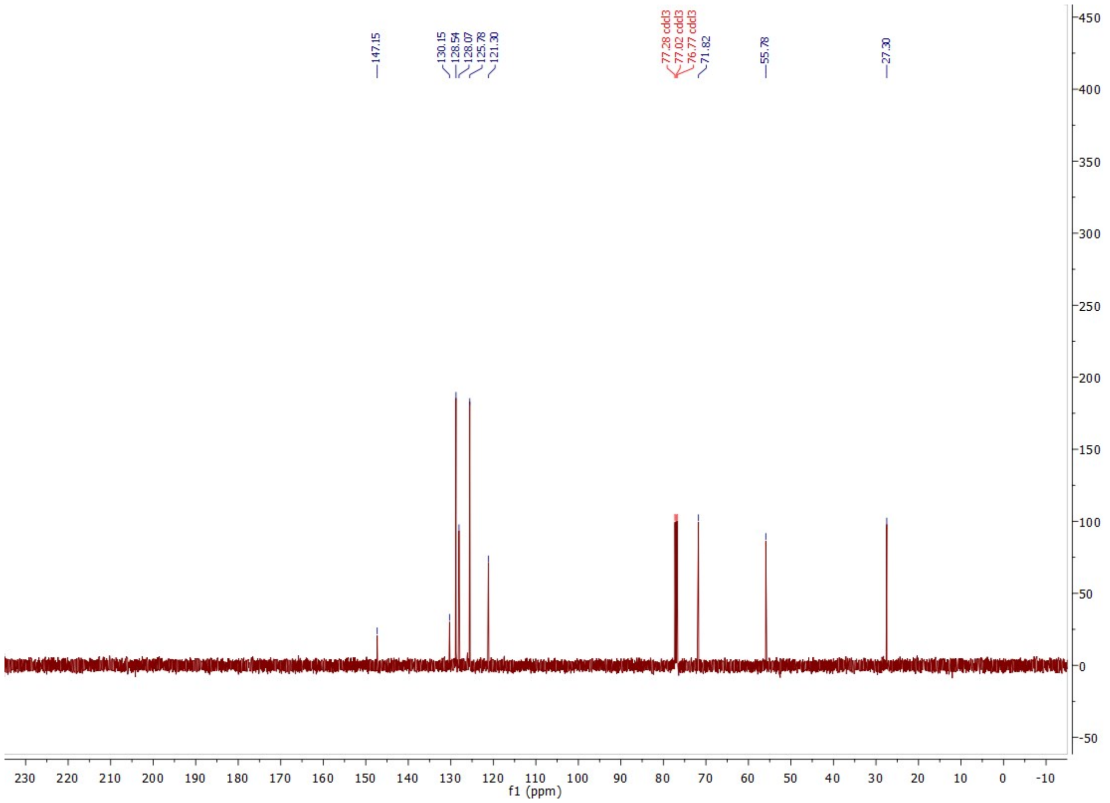

Figure S28. ^{13}C NMR Spectrum of (1-(2-mercapto-1-phenylethyl)-1*H*-1,2,3-triazol-4-yl)methanol (10b)

2-(4-(4-Methoxyphenyl)-1H-1,2,3-triazol-1-yl)-2-phenylethane-1-thiol (11b)

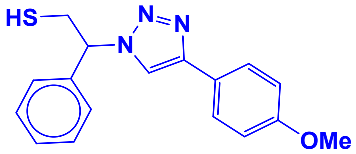

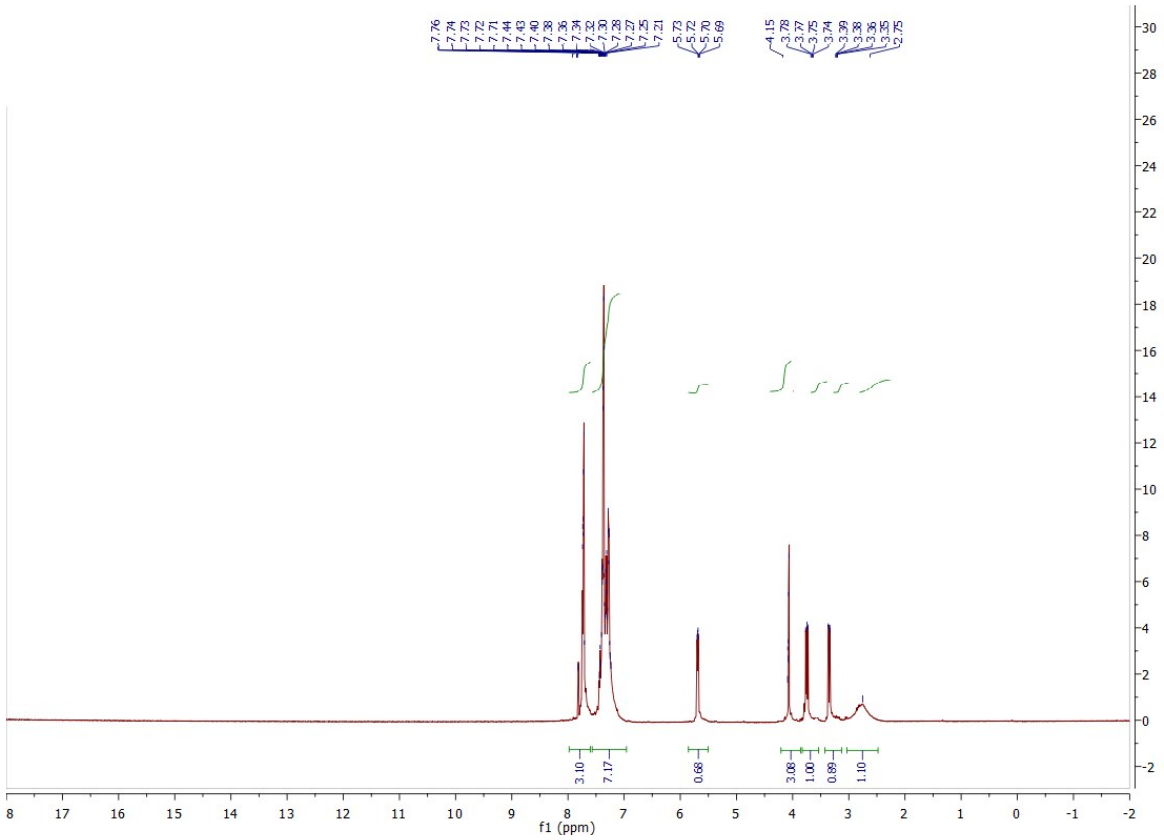

Figure S29. **¹H NMR Spectrum of 2-(4-(4-methoxyphenyl)-1*H*-1,2,3-triazol-1-yl)-2-phenylethane-1-thiol (11b)**

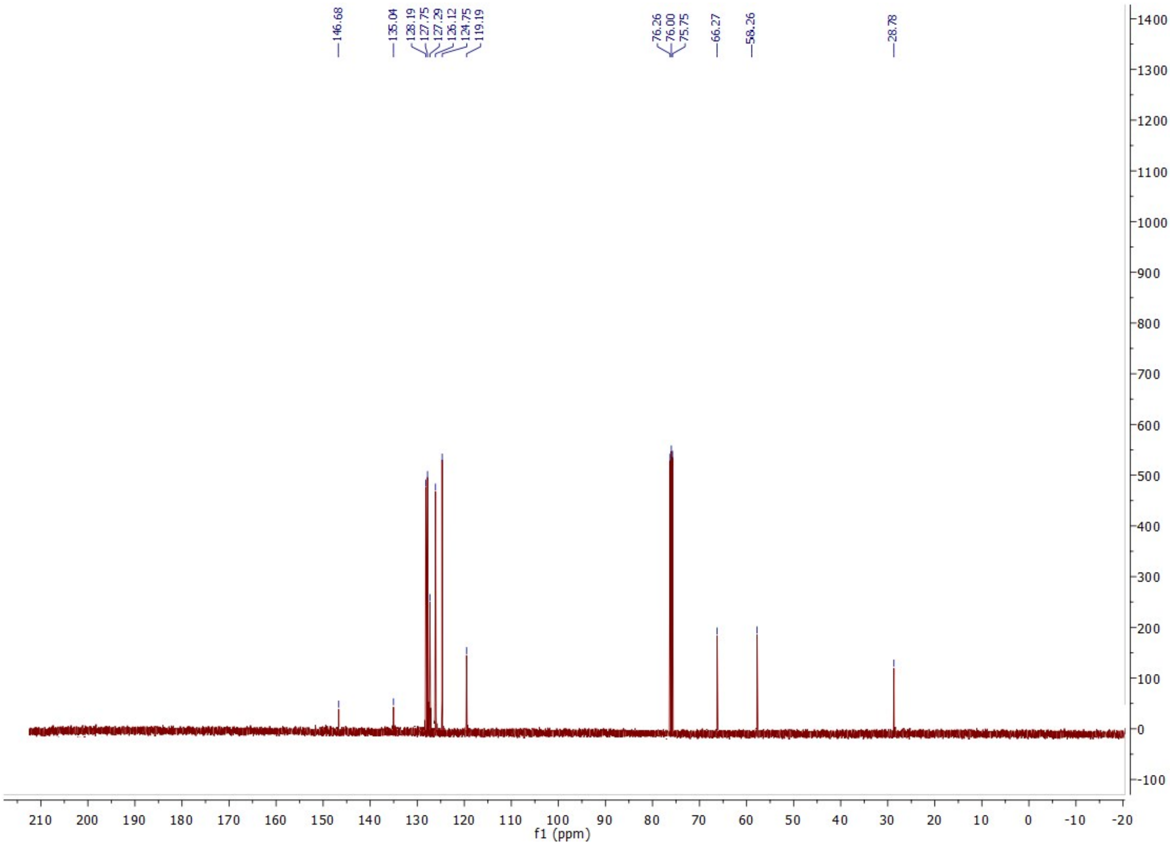

Figure S30. ^{13}C NMR Spectrum of 2-(4-(4-methoxyphenyl)-1*H*-1,2,3-triazol-1-yl)-2-phenylethane-1-thiol (11b)

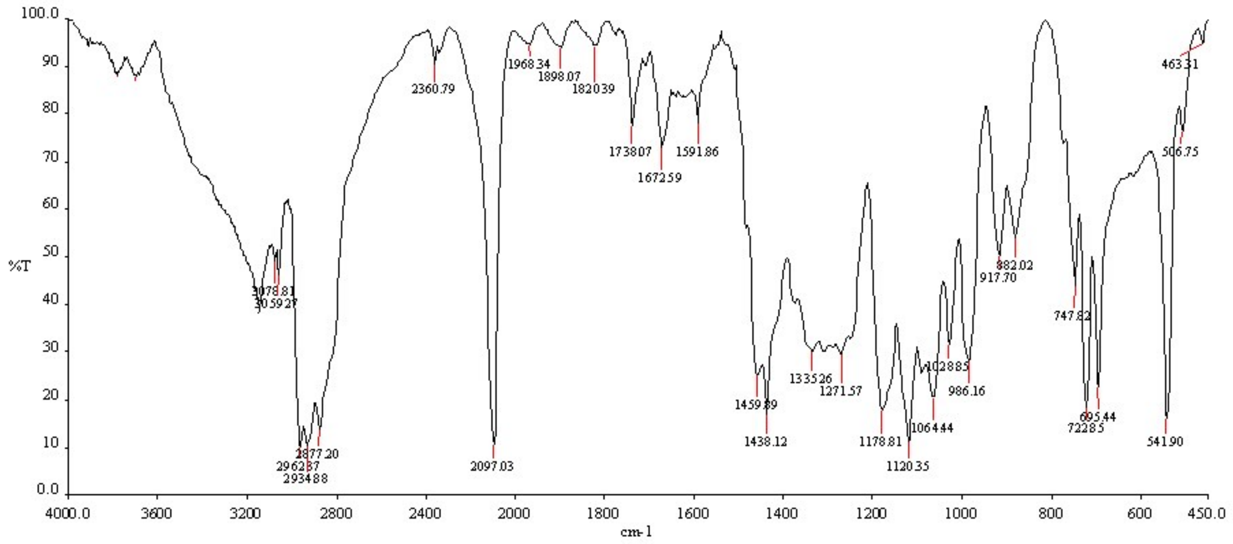

Figure S31. FT-IR Spectrum of 2-azido-2-phenylethanthiol intermediate
